# Engineered probiotic ameliorates hyperlipidemia and atherosclerosis by secreting PCSK9 nanobodies and regulating gut microbiota

**DOI:** 10.1002/btm2.70076

**Published:** 2025-09-19

**Authors:** Chuan Wang, Junyue Xing, Huan Zhao, Xiru Chen, Zongfeng Niu, Xiaohan Ma, Yuesheng Gui, Xinkun Qi, Yingchao Shi, Xiaolei Cheng, Dongdong Jian, Chao Shi, Hao Tang, Zhen Li

**Affiliations:** ^1^ National Health Commission Key Laboratory of Cardiovascular Regenerative Medicine, Central China Subcenter of National Center for Cardiovascular Diseases, Henan Cardiovascular Disease Center, Fuwai Central‐China Cardiovascular Hospital Central China Fuwai Hospital of Zhengzhou University Zhengzhou China; ^2^ Zhengzhou Key Laboratory of Cardiovascular Aging Central China Fuwai Hospital of Zhengzhou University Zhengzhou China; ^3^ Shanghai Xuhui Central Hospital Zhongshan‐Xuhui Hospital, Fudan University Shanghai China; ^4^ Henan Key Laboratory of Chronic Disease Management Central China Fuwai Hospital of Zhengzhou University Zhengzhou China; ^5^ Department of Oncology The First Affiliated Hospital of Zhengzhou University Zhengzhou China; ^6^ Henan Key Laboratory of Molecular Pathology, Department of Molecular Pathology The Affiliated Cancer Hospital of Zhengzhou University Zhengzhou China

**Keywords:** atherosclerosis, engineered probiotic, hyperlipidemia, nanobody, orally delivered, PCSK9

## Abstract

Elevated levels of low‐density lipoprotein cholesterol (LDL‐C) play a critical role in the onset and progression of cardiovascular disease (CVD). Inhibitors or monoclonal antibody drugs targeting pro‐protein convertase subtilisin/kexin type 9 (PCSK9) are novel cholesterol‐lowering medications that can effectively reduce serum LDL‐C levels. However, these drugs are usually expensive and require injections, which can reduce patient compliance and increase the financial burden. In this study, we constructed an engineered probiotic strain containing a prokaryotic expression element and a high‐affinity fragment of the human PCSK9 nanobody (PCSK9nb). The engineered bacterium was evaluated in vitro and in vivo for its ability to express and release PCSK9nb, as well as for its biocompatibility and stability. The therapeutic potential of the engineered probiotics was confirmed using mouse models of hyperlipidemia and atherosclerosis. We analyzed differences in mouse gut microbiota using high‐throughput sequencing and compared the therapeutic efficacy of the engineered bacteria with that of atorvastatin in a mouse model of hyperlipidemia. The engineered bacteria were found to express and release PCSK9nb in vivo after oral administration, achieving the effect of lowering serum cholesterol levels, alleviating atherosclerosis, and reducing body weight. In vivo, PCSK9nb was found to increase hepatic LDL receptor (LDLR) expression levels, decrease serum LDL‐C content, regulate the diversity and community structure of gut microbiota, reduce lipid accumulation in the liver, and decrease systemic inflammation. By comparing their efficacy with that of statins, the engineered probiotics demonstrated similar therapeutic effects. The research results provide a new strategy for the development of orally delivered PCSK9 antibody drugs, reducing healthcare costs and minimizing statin drug tolerance.

**Translational Impact Statement**

In this study, we developed a novel bacterial drug delivery system targeting PCSK9. By constitutively expressing a high‐affinity fragment of human PCSK9 nanobody in intestinal probiotics, we achieved oral delivery and in situ expression of PCSK9 nanobodies in the gut. Experiments confirmed that the secreted PCSK9 nanobody from the engineered bacteria effectively reduced serum LDL‐C levels, achieving therapeutic effects comparable to statin drugs. This approach may offer a novel alternative for the clinical use of lipid‐lowering medications in the future.

**Abbreviations**
ASCVD: atherosclerotic cardiovascular disease
CFU: colony forming units
CHOL: total cholesterol
CVD: cardiovascular disease
EcN: *Escherichia coli* Nissle 1917
EcN/PCSK9nb: recombinant engineered bacterium
HDL‐C: high‐density lipoprotein cholesterol
HFD: high‐fat diet
LDL‐C: low‐density lipoprotein cholesterol
PCSK9: pro‐protein convertase subtilisin/kexin type 9
PCSK9nb: the camelid single‐domain antibody VHH‐B11
VHH: heavy‐chain single‐domain antibody with a highly variable region

## 1 INTRODUCTION

Cardiovascular disease (CVD) is the primary cause of mortality worldwide.^1^, ^2^, ^3^ Elevated levels of low‐density lipoprotein cholesterol (LDL‐C) have been identified as important contributing factors in atherosclerosis and coronary heart disease.^4^ Proprotein convertase subtilisin/kexin type 9 (PCSK9) is a secreted protein synthesized in the endoplasmic reticulum of liver cells. It is involved in various mechanisms that promote atherosclerotic CVD (ASCVD), such as by increasing LDL‐C levels and exacerbating inflammatory reactions.^5^, ^6^, ^7^ The inhibition of PCSK9 can substantially reduce serum lipid levels and improve the occurrence or progression of ASCVD. Thus far, the development of therapeutic drugs targeting PCSK9 has become an important direction for drug research and development, and the results are expected to be widely used in metabolic diseases or tumors based on improving LDL‐C levels.^8^, ^9^, ^10^

PCSK9 inhibitors are a novel class of cholesterol‐lowering drugs that can substantially reduce serum lipid levels. Current, clinically approved PCSK9‐inhibitors include amorobumab, evolocumab, and tolesizumab, which are used in patients with ASCVD who are on maximum tolerated statin therapy.^11^ However, PCSK9 inhibitors have adverse effects. Research indicates that in hospital registries, 41.5% of patients report adverse events (AE), with injection site reactions (33.8%) and flu‐like symptoms (27.9%) being the most common.^12^ Muscle pain is the most frequently reported AE in drug surveillance databases. Inclisiran, a small interfering RNA (siRNA) PCSK9 therapy, also received approval in 2021.^13^ Compared with monoclonal antibody drugs, it has a relatively longer duration of action, but the therapeutic efficacy is below expectations. Long‐term adverse reactions remain unclear.^14^ Considering the common disadvantages of PCSK9 inhibitor drugs, such as high unit cost, injectable administration, shorter treatment cycle, and high economic burden, all of which may lead to low patient compliance, the development of an inexpensive, safe, stable, orally available, novel PCSK9 inhibitor has become increasingly important.

Nanobodies, a camelid‐derived, heavy‐chain single‐domain antibody with a highly variable region (VHH), are considered an attractive alternative to immunoglobulins (IgG) because of their nanoscale size (molecular weight of only ~15 KD) and ability to cross the blood–brain barrier.^15^, ^16^ Nanobodies are expressed not only in yeast but also in prokaryotes such as bacteria.^17^, ^18^ The high affinity, specificity, and stability of nanobodies make them superior to traditional antibodies and superior in disease diagnosis and treatment.^16^, ^19^, ^20^ Recently, Li et al. obtained PCSK9 nanoantibodies from alpacas using phage display technology and screened the effective binding region, VHH–B11.^9^ These nanobodies have shown potential in the preparation of PCSK9nb, although a mature treatment method for PCSK9nb has yet to be established. The first nanobody‐based drug, caplacizumab, was approved by the European Medical Agency (EMA). It is a bivalent VHH designed to treat thrombotic thrombocytopenic purpura and thrombosis.^21^

Microbial therapies that use engineered microorganisms as drug delivery systems for the targeted release of therapeutic agents in vivo have become a hotspot of attention in recent years owing to favorable characteristics, including oral administration, low cost, and the minimization of systemic side effects.^22^ Biologically active drugs using intestinal commensal microorganisms as vectors have been developed and applied for the diagnosis and treatment of a variety of diseases, such as metabolic disorders,^23^, ^24^, ^25^ autoimmune diseases,^26^, ^27^ hypertension,^28^ and certain solid tumors.^29^, ^30^, ^31^ *Escherichia coli* Nissle 1917 (EcN) is a commonly used bacterial substrate with a good genetic background, a short growth cycle, easy cultivation, and low production cost.^32^ EcN has been widely used as a probiotic of human intestinal origin and has been shown to be effective in the treatment of various gastrointestinal disorders. Engineered EcN strains have shown promising experimental results in ameliorating phenylketonuria, obesity, and type 2 diabetes in mice.^33^, ^34^, ^35^ Although not yet used in clinical practice, the use of engineered microorganisms for biotherapeutic purposes is still considered a promising direction for research and development with prospects for clinical application in metabolic disorder therapy.

In this study, we engineered the human probiotic EcN to express a PCSK9 nanobody, aiming to enable oral delivery of PCSK9 inhibitors with sustained intestinal release, enhanced stability, and improved safety. This approach provides a novel strategy and theoretical foundation for developing next‐generation PCSK9‐inhibiting therapeutics.

## 2 MATERIALS AND METHODS

### 2.1 Genetic engineering strain construction and bacteria culture conditions

The amino acid sequences for the camelid single domain antibody (sdAb) VHH‐B11 were obtained from Table S1 of a publication. According to previous literature, VHH‐B11 sdAb (hereafter referred to as PCSK9nb) exhibits the highest affinity toward human PCSK9 (hPCSK9). Nucleotide sequence optimization and synthesis of PCSK9nb was performed by GENEWIZ. In addition, a *tac* promoter, a ribosome binding site (RBS), and four signal peptides (NSP4, *dsbA*, *pelB*, and *phoA*) *were* respectively inserted sequentially into the 5′ end of PCSK9nb nucleic acid sequence to initiate its transcription, ribosome binding, and promote the expression and secretion of fusion proteins. A hemagglutinin (HA) antigen tagging peptide (HA‐tag) is inserted at the 3′ end of the PCSK9nb sequence as a tag to facilitate subsequent detection. *Hind* III and *BamH* I restriction enzyme sites were added before and after the fragment for recombination. The synthesized nucleotide sequence was then cloned into the pUC19 vector to generate the recombinant plasmids pUC19‐*tac*‐(NSP4/*dsbA*/*pelB*/*phoA*)‐PCSK9nb. The constructed recombinant plasmid was added to *E. coli* Nissle 1917 (EcN) competent cells, incubated on ice for 30 min, then transferred to a 2‐mm electroporation cuvette, placed in an electroporator, and subjected to an electric shock at 12.5 KV/cm for 5 ms. After pulsing, 1 mL of liquid LB medium was quickly added to the cells and gently mixed. The mixture was then placed on ice in a 1.5 mL EP tube. After incubation at 37°C and 200 rpm/min for 1 h, 200 μL was spread on an LB solid plate containing ampicillin and left to stand overnight at 37°C for screening to obtain the recombinant engineered bacterium EcN/pUC19‐*tac*‐(NSP4, *dsbA*, *pelB*, or *phoA*)‐PCSK9nb. To validate and screen the optimal signal peptides for PCSK9nb expression, a Western blot (WB) assay was performed through using anti‐HA tag antibody (Proteintech, 51064‐2‐AP). EcN and recombinant engineered strains were cultured in 5 mL LB medium with shaking (200 rpm) at 37°C overnight; 1 mL cultures were harvested by centrifuging (10,000*g*, 5 min, 4°C), and the cell‐free supernatant and precipitate were collected for WB.

### 2.2 PCSK9nb expression, growth curve test, and probiotic properties assessment

To establish the necessity of the NSP4 signal peptide for PCSK9nb extracellular secretion, isogenic recombinant strains harboring or lacking NSP4 were engineered (EcN/PCSK9nb and EcN/PCSK9nb [NSP4‐]), followed by WB validation of secreted (supernatant) and intracellular (precipitate) protein fractions.

Growth curve was performed on Micro‐GCM (BMG LABTECH). Briefly, single colonies of EcN and EcN/PCSK9nb were picked from solid LB plates into 5 mL liquid LB medium, cultured until the bacterial concentration reached ~2 × 10^8^ CFU/mL (OD₆₀₀ ≈ 2.0). Then 10 μL of the stationary‐phase culture was reinoculated into six‐well plates containing 3 mL LB medium per well, followed by shaking incubation at 37°C, 200 rpm. OD₆₀₀ was monitored every 30 min for a total of 20 h. The growth curves were created from at least three independent measurements using GraphPad Prism 8.0.

To measure the survival time of bacteria in the body, six mice were divided into two groups and given a dose of 10^8^ CFU EcN/PCSK9nb by gavage. Feces were continuously collected one to 4 days after gastric administration, resuspended in PBS and diluted, and then spread on ampicillin‐resistant LB (LBA) solid medium. After overnight culture at 37°C, the number of colonies was observed, plate photos were counted using ImageJ software, and statistics were performed with GraphPad Prism 8.0. Colonies were randomly selected for PCR identification, and the upstream and downstream parts of the PCSK9nb fragment were selected as primers.

### 2.3 Tolerance to Artificial Gastric Juice and Bile Salts

To determine the tolerance of the engineered strain EcN/PCSK9nb to simulated gastric fluid and bile salts, we performed in vitro culture analysis following previously established protocols.^36^ Briefly, freshly cultured cells were centrifuged at 6000*g* for 2 min and washed twice with sterile saline. The bacterial pellets were resuspended in simulated gastric fluid (LBA broth, pH 2.5, supplemented with 1% [w/v] pepsin) and incubated at 37°C for 4 h. Subsequently, the pellets were harvested, washed twice with sterile saline, and re‐suspended in LBA broth containing 0.4% (w/v) bile salts (Aladdin, B485289). After 12 h incubation at 37°C, serial dilutions of the bacteria were plated onto LBA agar for colony counting.

### 2.4 Animal model experiments under high‐fat diet

Six to eight‐week‐old male C57BL/6J or *Apoe−/−* mice weighing approximately 18–20 g were purchased from Beijing Sibeifu Experimental Animal Co., Ltd. These mice were housed in a specific pathogen‐free (SPF) environment under a 12‐hour light/dark cycle at a constant temperature (24 ± 2°C) and humidity.

Animal models of hyperlipidemia or atherosclerosis were made using mice fed a high‐fat diet (HFD) (Research Diets, D12492). Mice were gavaged with either PBS, EcN/pUC19, or EcN/PCSK9nb (1 × 10^9^ CFU in 200 μL of PBS) every 3 days, respectively.

The weight was assessed weekly, and blood was collected for further research once per month. Then, the animals were sacrificed after intraperitoneal injection of 1.5% isoflurane (RWD, R510‐22‐10). Colon, ileum, jejunum, and other tissues, such as heart, liver, spleen, lung, and kidney tissues, were collected after sacrifice and stored partly at −80°C and partly in 4% paraformaldehyde for subsequent experiments.

### 2.5 Immunohistochemistry and immunofluorescence

IHC and IF experiments were performed following standard procedures. Liver tissue was immersed in 4% paraformaldehyde (biosharp, BL539A), cut into 5‐μm‐thick sections, de‐paraffinized in xylene, and rehydrated in a graded alcohol series. The sections were then incubated with 5% goat serum for 1 h at room temperature. For IF, rabbit anti‐HA tag (Proteintech, 25,859‐1‐AP) was used as the primary antibody. Cy3‐conjugated anti‐rabbit antibody (Servicebio, GB21303) was used as the secondary antibody.

### 2.6 Oil red O and Nile red staining

Fix the isolated mouse heart or liver tissue block in 4% formaldehyde overnight. After fixation, remove the tissue and dehydrate in a 30% sucrose solution at 4°C. Place the tissue in a mold, add an appropriate amount of OCT compound (SAKURA, 4583), and freeze at −80°C. Use a cryostat to section the tissue. The thickness of the sections should be 10 μm. Allow the frozen sections to thaw at room temperature for 10 minutes. Prepare the Oil Red O staining (Jiancheng Biotech, D027‐1‐2) solution as needed. Immerse the sections in a staining dish containing PBS to remove excess OCT compound, then soak in distilled water (ddH_2_O) for 5 min. Once the sections are completely dry, add the Oil Red O staining solution onto a glass slide, ensuring all tissue areas are covered. After staining for 12 min, gently rinse the slide and soak the sections in ddH_2_O at 37°C for 1 min. Stain the samples with hematoxylin for 2 min, rinse with water for 1 min, and then apply an aqueous mounting medium to the tissue on the slide. Under a microscope, lipid droplets or patches should appear red, while cell nuclei should be stained blue. Capture and collect images. Finally, quantitatively analyze the area of the patches using ImageJ software.

### 2.7 Hematoxylin and Eosin (H&E) staining

Liver tissues were fixed in 4% paraformaldehyde, dehydrated, and embedded in paraffin. Tissue slices were then dewaxed, hydrated, and stained with hematoxylin and eosin (H&E) (Beyotime, C0105S) to assess morphology.

### 2.8 Cholesterol and lipid level analysis

Fresh blood of mice was collected and serum was separated by centrifugation, and the level of total cholesterol (CHOL) (Applygen, E1003), low density lipoprotein (LDL‐C) (Applygen, E1018), and high‐density lipoprotein (HDL‐C) (Applygen, E1017) was detected by the detection kit method. Calculate the concentration of cholesterol based on the standard curve using the optical density value of the sample tube.

### 2.9 Western blot

Liver tissues and colon tissues were homogenized. Tissues were lysed by RIPA lysis solution (Beyotime, P0013B) containing protease inhibitors (Solarbio, R0010), and then proteins were obtained by centrifugation at 12,000 rpm for 30 min at 4°C. Protein concentrations were quantified by BCA kit (Thermo Fisher, A53226), then separated by SDS‐PAGE on 10%–12% gel and electro‐transferred to polyvinylidene difluoride membranes (Millipore, IPVH00010). After blocking with 5% skim milk at room temperature for 1 h, the membranes were then incubated with the specific primary antibodies, including rabbit anti‐GAPDH (CST, 174S). After washing more than three times, the membranes were incubated with goat anti‐rabbit antibody (Abcam, 205718) or goat anti‐mouse antibody (Abcam, 97265) for 2 h at room temperature. Finally, those specific proteins were detected by using a chemiluminescence solution (Thermo Fisher, 32209).

### 2.10 Co‐immunoprecipitation

Protein was extracted and quantified by homogenization of mouse‐derived hepatocytes (Hepa1‐6) using co‐immunoprecipitation (Co‐IP) buffer containing 1% NP‐40 (Solarbio, N8032) and protease inhibitors (Solarbio, R0010). Pipette 20 μL of beads into a clean 1.5 mL EP tube according to the experimental protocol. Add 1 mL of Co‐IP buffer, gently pipette the solution to disperse the magnetic beads (Vazyme, PB101) evenly, and incubate to prepare the magnetic bead‐antibody complex. The above complex was allowed to stand on a magnetic rack, eluted using Co‐IP buffer, heated to separate the magnetic beads from the proteins, and the proteins were collected for WB validation.

### 2.11 RNA preparation and RT‐qPCR

Total RNA was extracted from liver tissues using TRIzol reagent according to the manufacturer's protocol (Invitrogen, 15596026). RNA degradation and contamination were assessed by 1% agarose gel. RNA concentrations were assayed by NanoDrop 2000 spectrophotometer (Thermo Fisher Scientific, Waltham, MA, USA). Genomic DNA was removed and then transcribed into cDNA using PrimeScript™ RT reagent Kit with gDNA Eraser (Takara, RR047A). The procedure of RT‐qPCR was carried out using TB Green Premix Ex Taq II kit (Takara, RR820A), and all reactions were performed using Biorad CFX96 Real‐Time PCR System (Biorad, CA, USA). The program of RT‐qPCR was 95°C for 30 s, followed by 40 cycles of 95°C for 5 s and then 60°C for 34 s. Primers for IL‐1β, IL‐6, TNF‐α, MCP‐1, and β‐Actin were listed in Table S2. The relative expression levels were measured using the 2^−ΔΔCt^ method.

### 2.12 16s rRNA sequencing

Mouse feces were collected, snap‐frozen in liquid nitrogen, and stored at −80°C until use. 16S rRNA amplicon sequencing and analysis were conducted by OE Biotechnology Co., Ltd. (Shanghai, China). Extract total genomic DNA using a DNA extraction kit following the manufacturer's instructions. Genomic DNA is used as a template for PCR amplification using barcoded primers and Tks Gflex DNA polymerase. For bacterial diversity analysis, universal primers 343F (5′‐TACGGRAGGCAGCAG‐3′) and 798R (5′‐AGGGTATCTAATCCT‐3′) were used to amplify the V3‐V4 variable region of the 16S rRNA gene. Raw sequencing data is in FASTQ format. Low‐quality sequences were filtered, denoised, merged, and chimeric sequences were detected and removed using the default parameters of DADA2 and QIIME2 (2020.11). The representative reads of each Amplicon Sequence Variant (ASV) were selected using the QIIME 2 packet. All representative reads of Silva database version 138 (or Unite) are annotated and blasted using q2‐feature‐classifier and default parameters.

### 2.13 Statistical analysis

The experimental results were scanned using ImageJ image editing software (National Institutes of Health, United States), analyzed using SPSS 23.0 (IBM Company, United States), and graphed using GraphPad Prism 8.0 (GraphPad Software Company, United States). For two‐sample quantitative data that follow a normal distribution, a two‐sample independent t‐test is used. For multi‐group sample statistics, one‐way ANOVA is employed. Data are expressed as mean ± standard deviation, and statistical significance is set at *p* < 0.05.

## 3 RESULTS

### 3.1 Construction of PCSK9 nanobody (PCSK9nb)‐engineered probiotics

To achieve prokaryotic expression and oral delivery of PCSK9nb, we codon‐optimized the amino acid sequence of the previously reported high‐affinity fragment, VHH–B11, of the human PCSK9 nanobody (hPCSK9nb) prepared from alpacas to facilitate its expression in a prokaryotic system (Table S1). BLAST sequence alignment analysis showed that the PCSK9 amino acid sequence was highly conserved between humans and mice, with an identity of 77.71% (Figure S1a). Analysis of the human and mouse PCSK9 protein domains on the SMART website (http://smart.embl-heidelberg.de/) revealed that both contain a peptidaseS8 domain that functions as a serine peptidase (Figure S1b). The difference between the two lies in the fact that, when compared with the predicted protein domain of mouse PCSK9, the 77–152 amino acid sequence of the human PCSK9 protein is annotated as an inhibitor_I9 domain, which is defined as a subtilisin peptidase. The N‐terminal propeptide inhibitor domain, the subtilisin propeptide, serves as a molecular chaperone that helps to regulate the folding and activity of mature peptidases.

The SWISS‐MODEL website was used to compare the tertiary structures of human and mouse PCSK9 proteins. With the human PCSK9 protein (Q8NBP7.1. A) as a reference, the similarity between the two was 77.96% (Figure S1c). The amino acid sequences, functional domains, and three‐dimensional structures of human and mouse PCSK9 are very similar, suggesting that they perform similar functions. This indicates that the VHH–B11 amino acid sequence of hPCSK9nb can be used in animal experiments for functional verification. The specific construction process of EcN/PCSK9nb engineered probiotics was carried out as described in the Materials and Methods section (Figure S1d).

**FIGURE 1:**
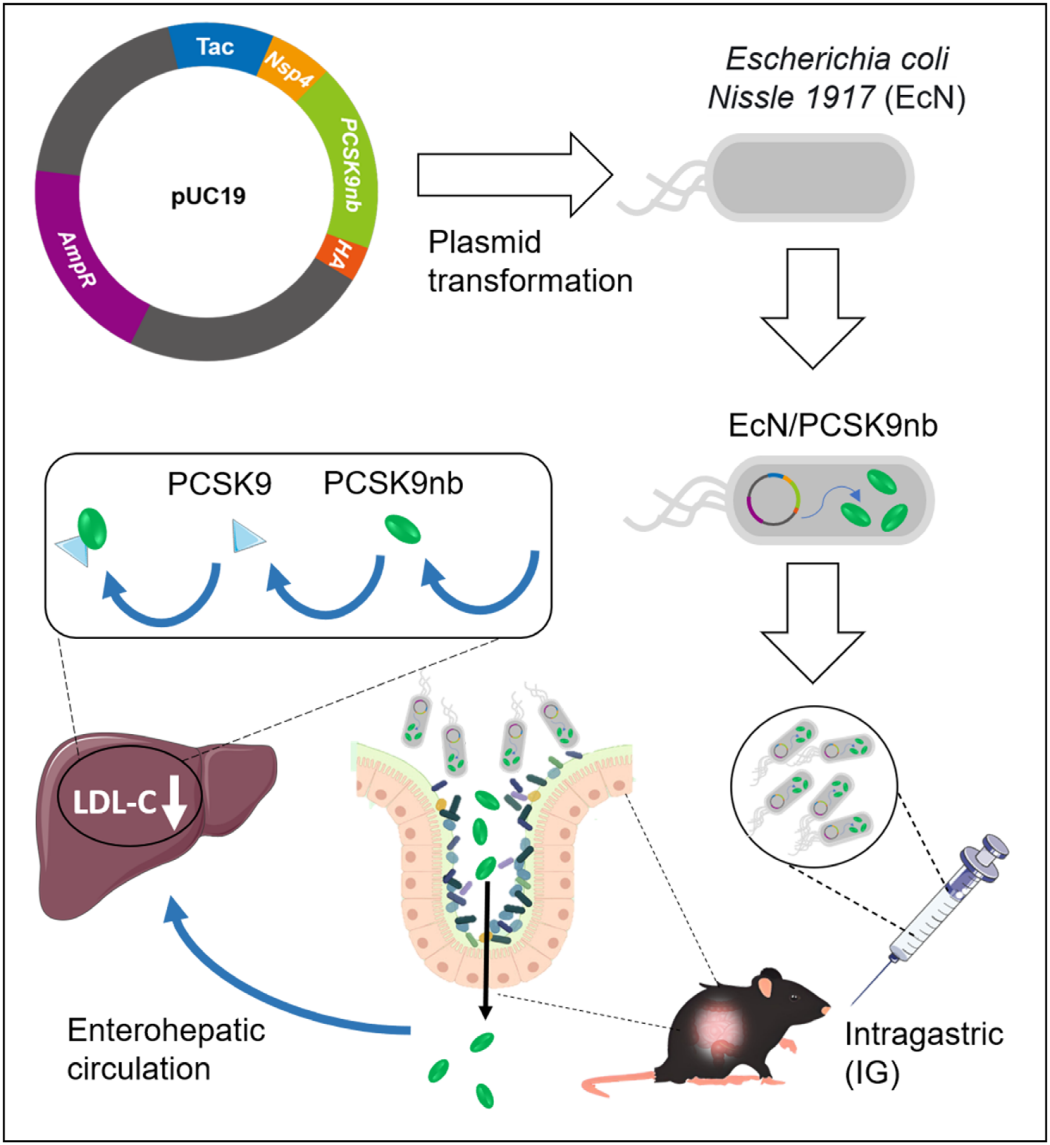
Engineering strategy and effect pathway of recombinant engineered probiotics.

The following research strategy was proposed for this project: a recombinant plasmid containing the prokaryotic expression element and codon‐optimized PCSK9nb VHH‐B11 sequence was constructed and transformed into the intestinal probiotic EcN to obtain the recombinant engineered probiotic. The engineered bacteria expressed and released PCSK9nb into the mouse intestine after oral administration. PCSK9nb penetrates the intestinal barrier, enters the liver, and circulates through the portal vein. This process further reduces the binding of PCSK9 to LDLR in the liver, thereby lowering the LDL‐C levels and alleviating atherosclerotic disease (Figure 1).

### 3.2 Screening of engineered strains for high‐efficiency PCSK9nb secretion and physiological traits

**FIGURE 2:**
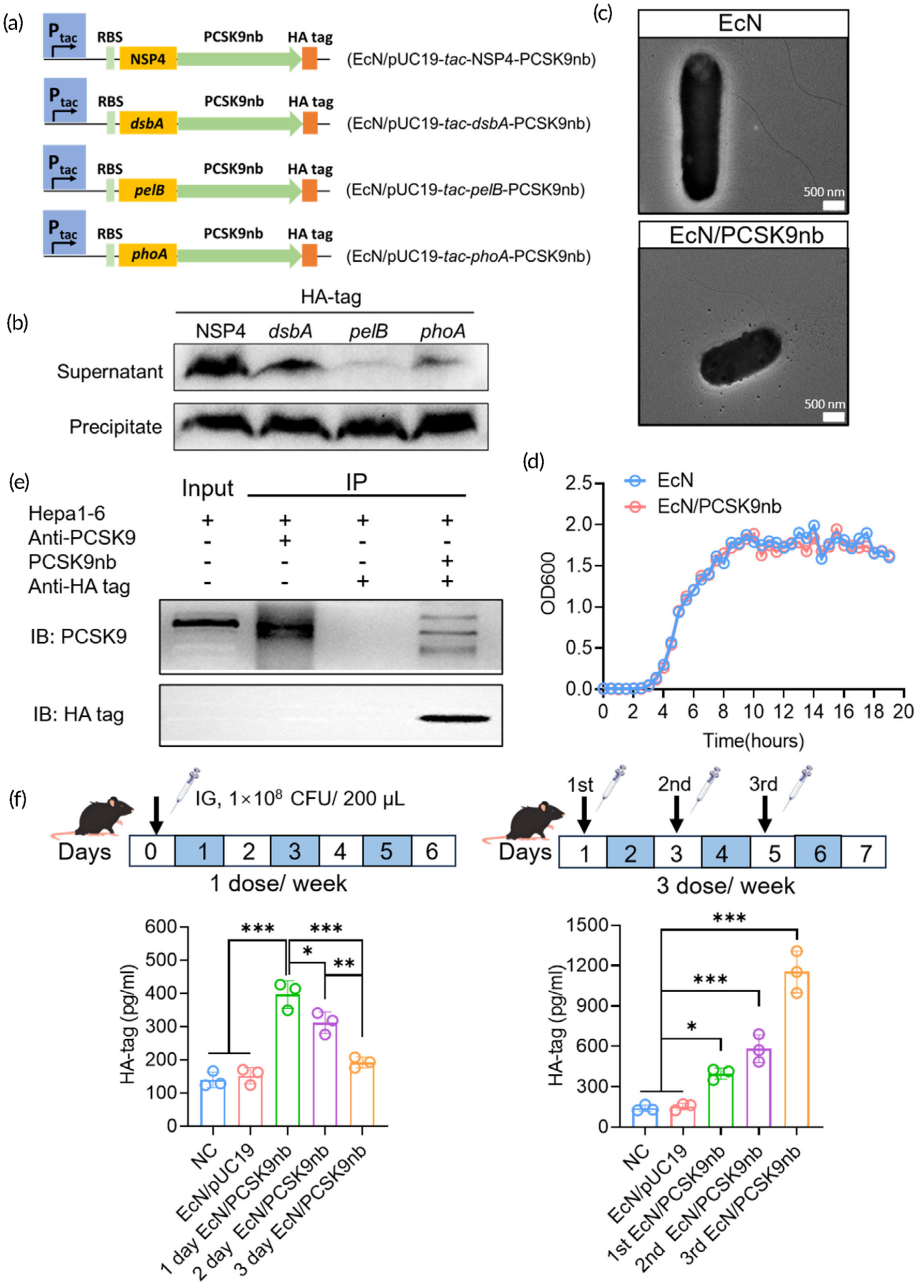
Screening of signal peptides in engineered strains and analysis of physiological characteristics. (a) Construction of engineered bacteria containing four signal peptides NSP4, *dsbA*, *pelB*, *phoA*; (b) Western blot analysis of culture supernatants and precipitate from four engineered strains by verifying the expression level of the HA tag in vitro; (c) Observation of the morphology of EcN and EcN/PCSK9nb strains using transmission electron microscopy, scale: 500 nm; (d) Growth curve of EcN and EcN/PCSK9nb; (e) Specific binding of the expressed and secreted PCSK9nb from EcN/PCSK9nb‐engineered bacteria to mouse PCSK9 was confirmed using Co‐IP experiments; (f) The serum levels of PCSK9nb in mice with single and multiple gavages of engineered bacteria were measured by ELISA. *p < 0.05, **p < 0.01, ***p <0.001.

To maximize secretory yield of PCSK9nb, four orthogonal signal peptides spanning viral (NSP4) and bacterial (*dsbA*, *pelB*, *phoA*) origins were incorporated into the expression chassis (Figure 2a). Western blot analysis of culture supernatants and precipitate from four engineered strains revealed that EcN/PCSK9nb carrying the NSP4 signal peptide achieved the most efficient secretion of PCSK9nb into the supernatant by verifying the expression level of the HA tag in vitro (Figure 2b). Concomitantly, we observed detectable expression of PCSK9nb in both supernatant and precipitate fractions of the NSP4 signal peptide‐containing strain. In contrast, PCSK9nb was exclusively detected in the precipitate of strains lacking the NSP4 signal peptide (Figure S2). These results demonstrate that efficient secretion of PCSK9nb requires guidance by the NSP4 signal peptide.

We observed the morphology of EcN/PCSK9nb using transmission electron microscopy (TEM), which indicated no obvious difference in morphology between the EcN/PCSK9nb engineered and original EcN strains (Figure 2c). The growth characteristics of the engineered bacteria, EcN/PCSK9nb, were evaluated by measuring their growth curves using micro‐GCM, which showed no difference in growth curves compared to the parental strain, EcN. This indicated that the recombinant plasmid containing PCSK9nb did not affect EcN growth (Figure 2d).

Co‐IP was performed to validate whether PCSK9nb secreted by the engineered bacterium, EcN/PCSK9nb, could bind specifically to PCSK9. Given the highly conserved amino acid sequence of PCSK9 in mammals, as indicated by previous sequence alignment analyses, it was theoretically plausible that PCSK9nb produced by EcN/PCSK9nb could selectively bind to mouse PCSK9. To investigate this, lysates from the mouse‐derived liver cell line, Hepa1–6, were incubated with magnetic beads conjugated with three different antibody combinations. The band in the fourth lane illustrates the successful binding of PCSK9nb to PCSK9 in the Hepa1–6 lysate (Figure 2e).

Next, the ability of EcN/PCSK9nb was used to express and secrete PCSK9nb normally in vivo. The level of PCSK9nb in the serum was determined using an enzyme‐linked immunosorbent assay (ELISA) with a single gavage (one dose/week) and multiple gavages of mice (three doses/week). After oral administration of EcN/PCSK9nb at one dose/week, the highest level of HA‐tag was detected in the serum on day 1 after gavage, followed by a gradual decrease. On day 3 after gavage, serum PCSK9nb levels showed no significant difference compared to the control group. In contrast, in mice receiving three doses/week, the level of detectable PCSK9nb in the serum increased progressively with the number of gavages, and the overall level was higher than that in mice gavage with a single dose (Figure 2f). These results suggest that EcN/PCSK9nb can efficiently secrete PCSK9nb in vivo and that periodic oral administration of PCSK9nb is required to maintain PCSK9nb concentrations in the serum during the intervention.

### 3.3 Colonization and distribution of EcN/PCSK9nb in vivo

**FIGURE 3:**
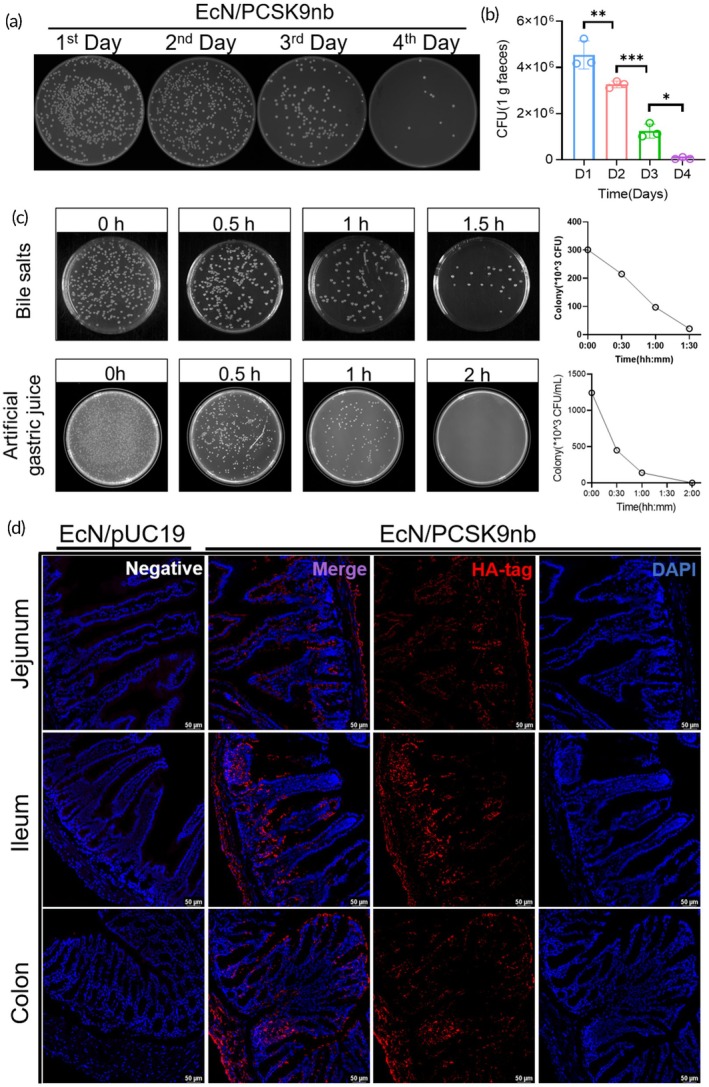
Analysis of the in vivo colonization ability of EcN/PCSK9nb. (a) The flat plate counting method was used to measure the in vivo residence time of *EcN/PCSK9nb*; (b) Statistical analysis was performed on the flat plate colony results, and the data was presented as mean ± SD (*n* = 3); (c) Validation of the tolerance of engineered bacterium EcN/PCSK9nb to artificial gastric juice and bile salts; (d) Immunofluorescence staining of the jejunum, ileum, and colon. Red represents the HA‐tag, blue represents DAPI, scale: 50 μm. The EcN/pUC19 treatment group served as the negative control. *p < 0.05, **p < 0.01, ***p < 0.001, ****p < 0.0001.

Next, the colonization ability of EcN/PCSK9nb in the mouse intestine was evaluated by counting the number of colonies in the feces. After administering a dose of 1 × 10^8^ colony‐forming units (CFU) of EcN/PCSK9nb, mouse feces were collected for four consecutive days. The collected feces were prepared as suspensions and uniformly coated onto lysogeny broth (LB) solid plates containing ampicillin (Amp). Under antibiotic pressure, the EcN/PCSK9nb strain grew normally on the Amp plate because it contained the AmpR gene element. However, other bacterial strains in mouse feces could not grow on the plates. We collected mouse feces of the same weight from days 1 to 4 after gavage with EcN/PCSK9nb. The feces were resuspended in equal volumes of PBS, diluted at the same dilution factor, and equal volumes of the fecal suspensions were plated on LBA plates. The results showed that the colony counts of EcN/PCSK9nb on the plates exhibited a significant decreasing trend over time (Figure 3a). By counting plate colonies, we calculated that the EcN/PCSK9nb content in feces was approximately 4 × 10^6^ CFU/g 1 day after intragastric administration and then gradually decreased. On the fourth day after intragastric administration, the EcN/PCSK9nb content was less than 1 × 10^5^ CFU/g (Figure 3b). We also confirmed that the randomly selected bacterial colonies in the four plates belonged to EcN/PCSK9nb. The results revealed a specific band at 520 bp, confirming that the colonies growing on the plate were all engineered bacteria EcN/PCSK9nb (Figure S3). Subsequently, we conducted artificial gastric juice and bile salt tolerance experiments on the engineered bacterium EcN/PCSK9nb in vitro. The results showed that EcN/PCSK9nb exhibited certain basic tolerance to artificial gastric juice and bile salt, maintaining bacterial viability within 1 hour or 1.5 hours after treatment. This is conducive to the bacteria entering the intestine and exerting their function in the intestinal tract (Figure 3c).

Additionally, we gavaged mice with EcN/PCSK9nb once every 3 days and harvested intestinal tissues 1 week after the first gavage. Immunofluorescence assay was used to detect the fusion expression of HA‐tag and PCSK9nb, confirming that the engineered strain EcN/PCSK9nb could normally express and secrete PCSK9nb in vivo. Staining results showed red fluorescence in the jejunum, ileum, and colon (Figure 3d). This indicates that PCSK9nb is secreted normally in the intestine. Furthermore, to investigate the distribution of PCSK9nb in extraintestinal organs after entering the circulation, the remaining tissues of the mice, including the heart, liver, spleen, lungs, and kidneys, were collected. Immunofluorescence staining results indicated that PCSK9nb could be detected in the liver, spleen, lung, and kidney after entering the circulation (Figure S4). In summary, EcN/PCSK9nb can colonize the intestine for a short period and secrete PCSK9nb into the circulation and other organ tissues.

### 3.4 EcN/PCSK9nb substantially reduces serum lipid levels in C57BL/6J mice

**FIGURE 4:**
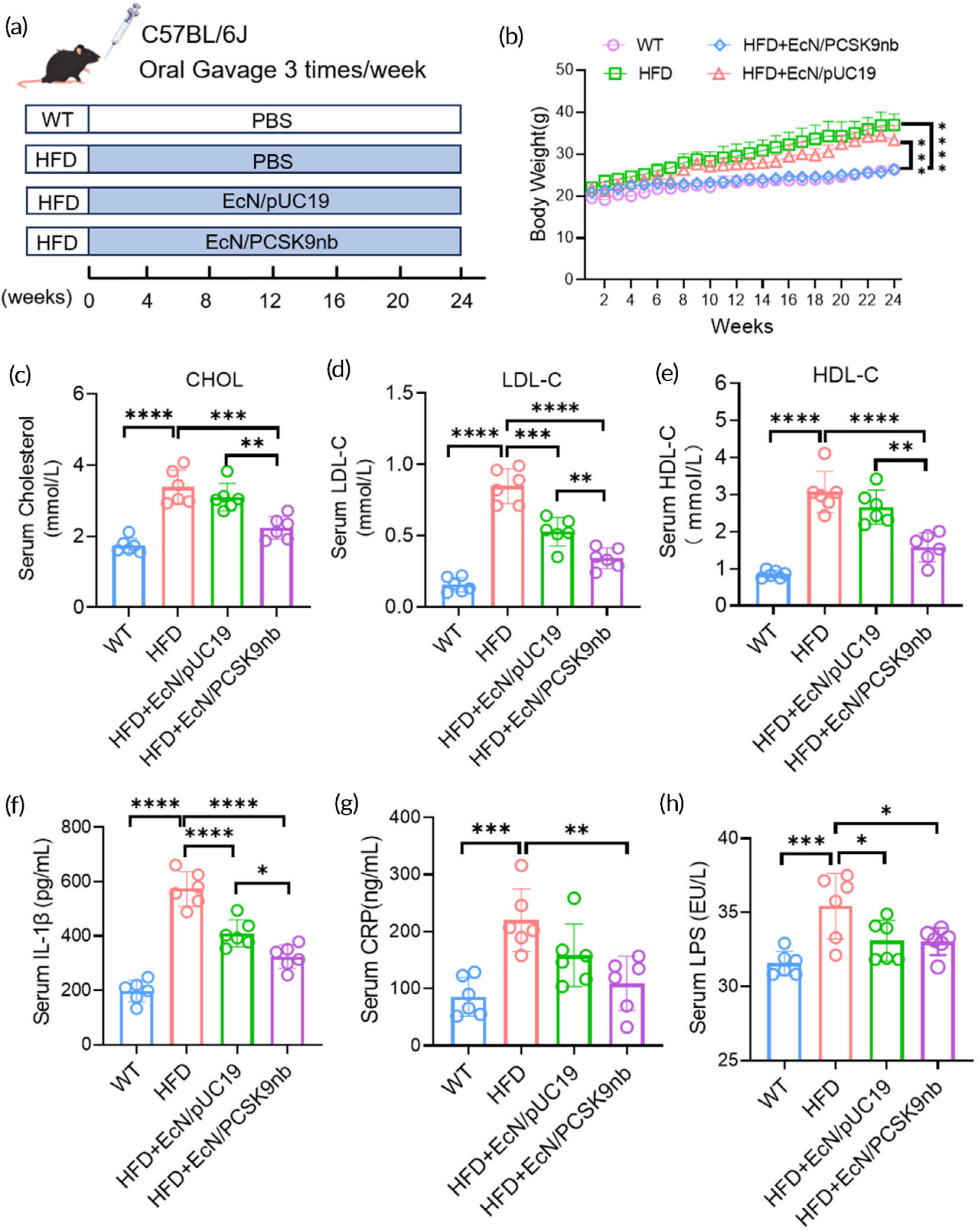
EcN/PCSK9nb substantially reduces serum lipid levels in C57BL/6J mice. (a) Experimental protocol for the hyperlipidemia model; (b) Body weight curves, statistical data presented as mean ± SD (*n* = 6); (c–e) Serum cholesterol, LDL‐C, and HDL‐c levels at 24 weeks; (f–h) Serum IL‐1β, CRP, and LPS levels at 24 weeks. Statistical data presented as mean ± SD (*n* = 6); **p < 0.01, ***p < 0.001, ****p < 0.0001.

Whether EcN could induce PCSK9nb to lower LDL‐C levels in vivo was tested by a HFD mouse model of hyperlipidemia. A total of 24, 8‐week‐old male C57BL/6J mice were divided into four groups with six mice in each group. All control and experimental groups were administered phosphate‐buffered saline (PBS), EcN/pUC19 (empty vector control), and EcN/PCSK9nb by oral gavage three times/week. Bacterial gavage doses were administered as described in the Materials and Methods (Figure 4a). Body weight changes were continuously monitored for 24 weeks. The results showed that the body weight gain of mice in the engineered bacterial EcN/PCSK9nb intervention group was significantly lower than that of HFD mice (Figure 4b). We collected serum from the mice at 4, 8, and 24 w to detect total cholesterol (CHOL), LDL‐C, and high‐density lipoprotein cholesterol (HDL‐C). At 4 weeks post‐treatment, although the serum total cholesterol level of the HFD group was higher than that of the wild‐type mice, there was no statistical difference in LDL‐C levels (Figure S5a). At 8 weeks post‐treatment, serum CHOL, LDL‐C, and HDL‐C levels began to increase substantially in the HFD group (Figure S5b), and these changes continued until the end of the 24‐week experiment. The mice administered EcN/pUC19 alone by gavage also showed a substantial decrease in serum LDL‐C levels at 24 weeks post‐treatment, but this decrease was not as marked as that in the EcN/PCSK9nb group (Figure 4c–e). Moreover, the levels of IL‐1β, CRP, and LPS in serum were analyzed by ELISA. The results showed that both were significantly alleviated in the EcN/PCSK9nb intervention group (Figure 4f–h).

### 3.5 EcN/PCSK9nb improves the atherosclerotic phenotype in *ApoE−/−* mice

**FIGURE 5:**
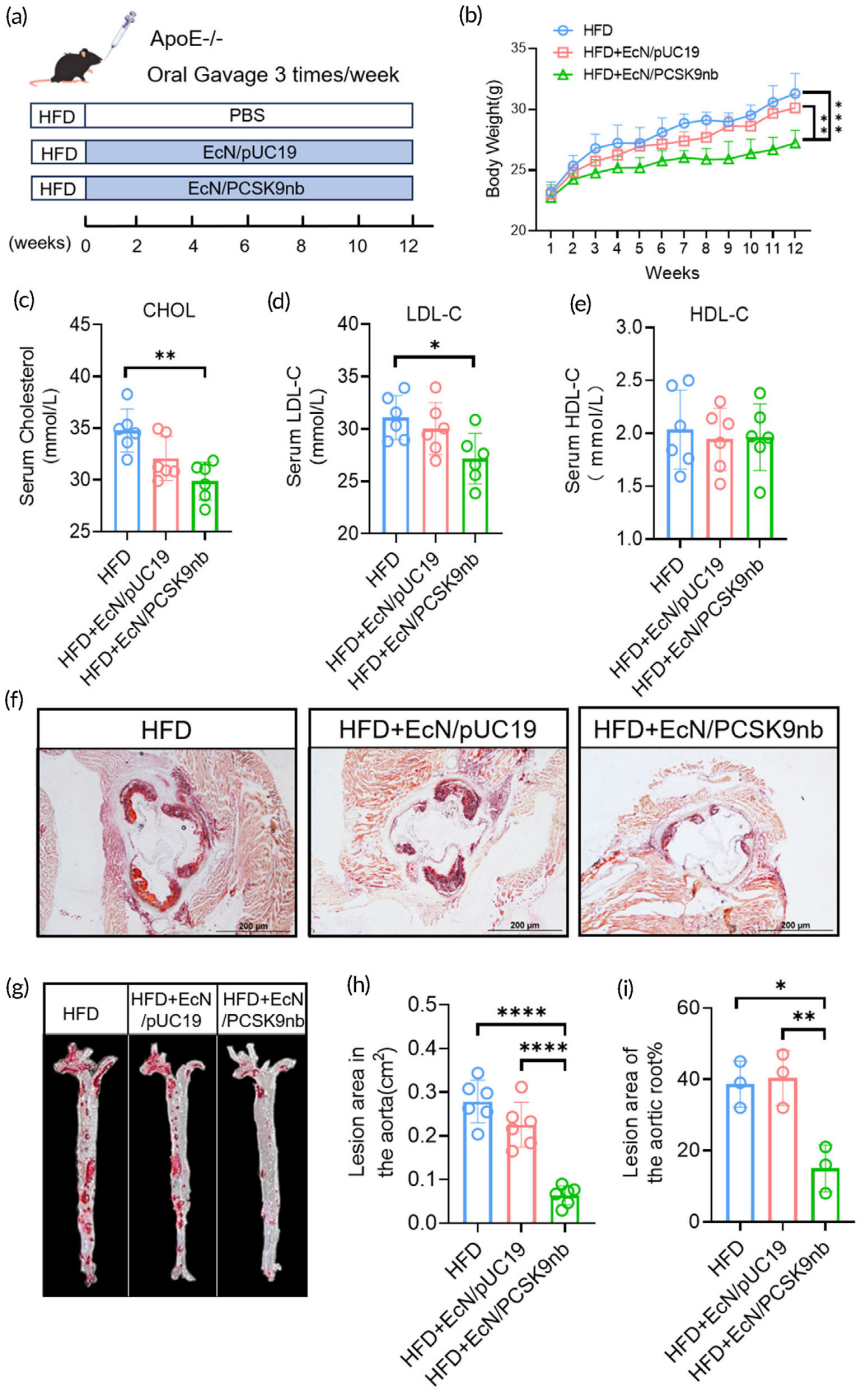
Oral administration of EcN/PCSK9nb can alleviate high‐fat diet‐induced atherosclerotic lesions. (a) Atherosclerosis model experimental plan; (b) Body weight curves, statistical data presented as mean ± SD (*n* = 6); (c–e) Serum CHOL, LDL‐C, and HDL‐C levels displayed as mean ± SD (*n* = 6); (f, i) Oil Red O staining and plaque area statistics of aortic root sections, with statistical data presented as mean ± SD (*n* = 3); (g, h) Oil Red O staining and plaque area statistics of the entire aorta, with statistical data presented as mean ± SD (*n* = 6). *p < 0.05, **p < 0.01, ***p < 0.001, ****p < 0.0001.

Furthermore, we used an atherosclerosis model using *ApoE−/−* mice to confirm whether EcN/PCSK9nb alleviated the phenotypes associated with atherosclerosis. Six‐week‐old male *ApoE−/−* mice were fed a high‐fat diet for 12 weeks to induce atherosclerosis. The mice were administered PBS, EcN/pUC19, or EcN/PCSK9nb by oral gavage three times a week (Figure 5a). The trend of weekly body weight change during the experiment was similar to that observed in the HFD‐induced hyperlipidemia model. EcN/PCSK9nb intragastric administration also substantially reduced the body weight of high‐fat fed *ApoE−/−* mice (Figure 5b). Serum total cholesterol (CHOL) and LDL‐C were substantially reduced in the EcN/PCSK9nb group of mice, but there was no significant difference in serum HDL‐C levels (Figure 5c–e). Oil Red O staining was performed on the aortic root sections to evaluate the atherosclerotic lesion areas. Compared to the HFD group mice that were gavaged with PBS, the mice in the EcN/PCSK9nb group showed fewer atherosclerotic lesions in the root sections. The plaque area was reduced substantially by 20% (Figure 5f,i). Oil Red O staining of the entire aorta showed that oral EcN/PCSK9nb substantially reduced the plaque area of atherosclerotic lesions compared to the HFD group (Figure 5g,h). PCSK9nb therefore delayed the development of atherosclerosis in *ApoE−/−* mice. These results suggest that oral administration of EcN/PCSK9nb can effectively alleviate HFD‐induced atherosclerotic lesions in *ApoE−/−* mice.

### 3.6 EcN/PCSK9nb reduces liver fat accumulation and inflammation

**FIGURE 6:**
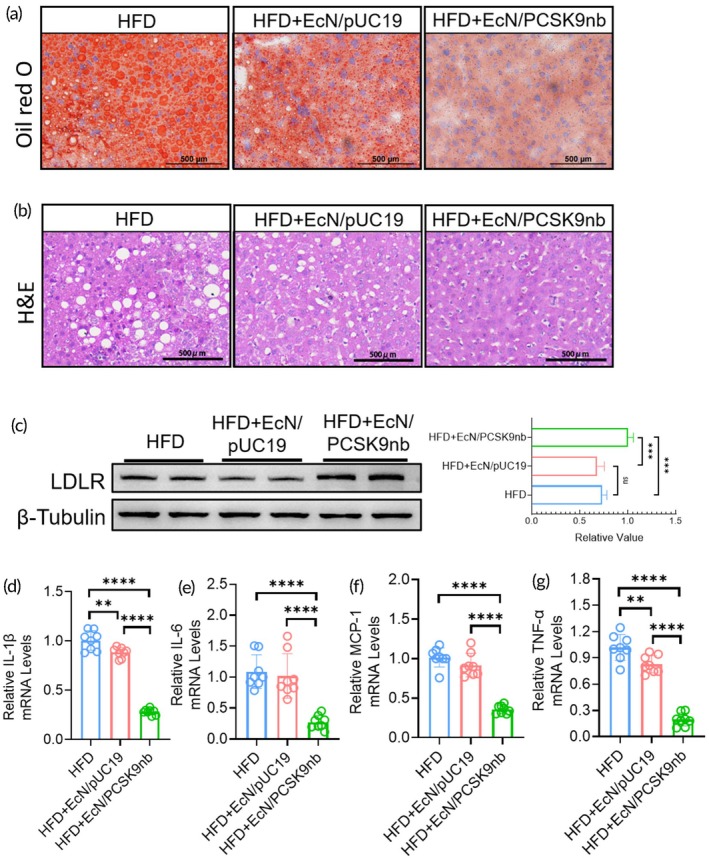
Engineered bacteria EcN/PCSK9nb reduce liver lipid accumulation, increase LDLR expression, and reduce inflammation levels. (a) Liver tissue section stained with Oil Red O, scale bar: 500 μm; (b) Liver tissue section stained with H&E, scale bar: 500 μm; (c) LDL‐R levels of C57BL/6J mice livers were evaluated by western blot; (d) qPCR analysis of transcription levels of inflammatory factors (IL‐1β, IL‐6, MCP‐1, and TNF‐α) in the liver. Statistical data presented as mean ± SD (*n* = 6). *p < 0.05, **p < 0.01, ***p < 0.001, ****p < 0.0001.

As mentioned previously, PCSK9nb mainly protects LDLR on the liver surface by neutralizing PCSK9, maintaining the ability of the liver to absorb lipids and stabilize serum lipids. Thus, we evaluated the ability of EcN/PCSK9nb to alleviate hepatic lipid accumulation through in vivo mouse experiments. Oil Red O staining was performed on liver sections of C57BL/6J mice fed a high‐fat diet for 24 weeks. Oral administration of EcN/PCSK9nb engineered bacteria can significantly alleviate liver fat accumulation induced by a high‐fat diet (Figure 6a and Figure S6a). Hematoxylin and eosin (H&E) staining of the mouse liver sections showed that the size and number of lipid droplet vacuoles were reduced in the EcN/PCSK9nb group (Figure 6b). These results consistently indicate that EcN/PCSK9nb engineered bacteria improve fat accumulation in the liver.

To verify the functional mechanism of PCSK9nb in vivo, western blot analysis was performed on the livers of C57BL/6J and *ApoE−/−* mice fed with a high‐fat diet. The oral administration of EcN/PCSK9nb engineered bacteria elevated hepatic LDLR levels in mice fed a high‐fat diet (Figure 6c and Figure S6b). This was due to the binding of PCSK9 to LDLR, thereby causing its degradation. PCSK9nb, secreted in vivo by engineered bacteria, enters the liver through the portal vein and binds to PCSK9, thereby increasing LDLR levels.

PCSK9 inhibitors can alleviate inflammation induced by high‐fat diets.^37^ Thus, we detected the mRNA levels of several inflammatory factors in liver tissues including interleukins 1β and ‐6, tumor necrosis factor (TNF)‐α, and monocyte chemoattractant protein 1 (MCP1) using real‐time quantitative polymerase chain reaction. The EcN/PCSK9nb treatment considerably alleviated the levels of liver inflammatory factors induced by the high‐fat diet (Figure 6d–g). These results suggest that EcN/PCSK9nb exerts its therapeutic effects against hyperlipidemia and atherosclerosis in mice and has the potential for the improvement of fatty liver disease.

### 3.7 EcN/PCSK9nb engineered bacteria improve HFD‐induced gut microbiota disorder

Numerous studies have shown that gut microbiota is one of the important factors affecting CVD and can improve metabolism‐related diseases, such as hyperlipidemia and atherosclerosis. Liver lipid accumulation in mice fed on a high‐fat diet was attenuated to some extent when EcN/pUC19 was administered orally (Figure 6a,b). In the current study, the chassis organism, EcN, was selected as a human intestinal‐derived probiotic. Therefore, it was necessary to analyze the diversity and community structure characteristics of gut microbiota caused by EcN/PCSK9nb engineered bacteria or parent strains, and to explore the role of gut microbiota.

**FIGURE 7:**
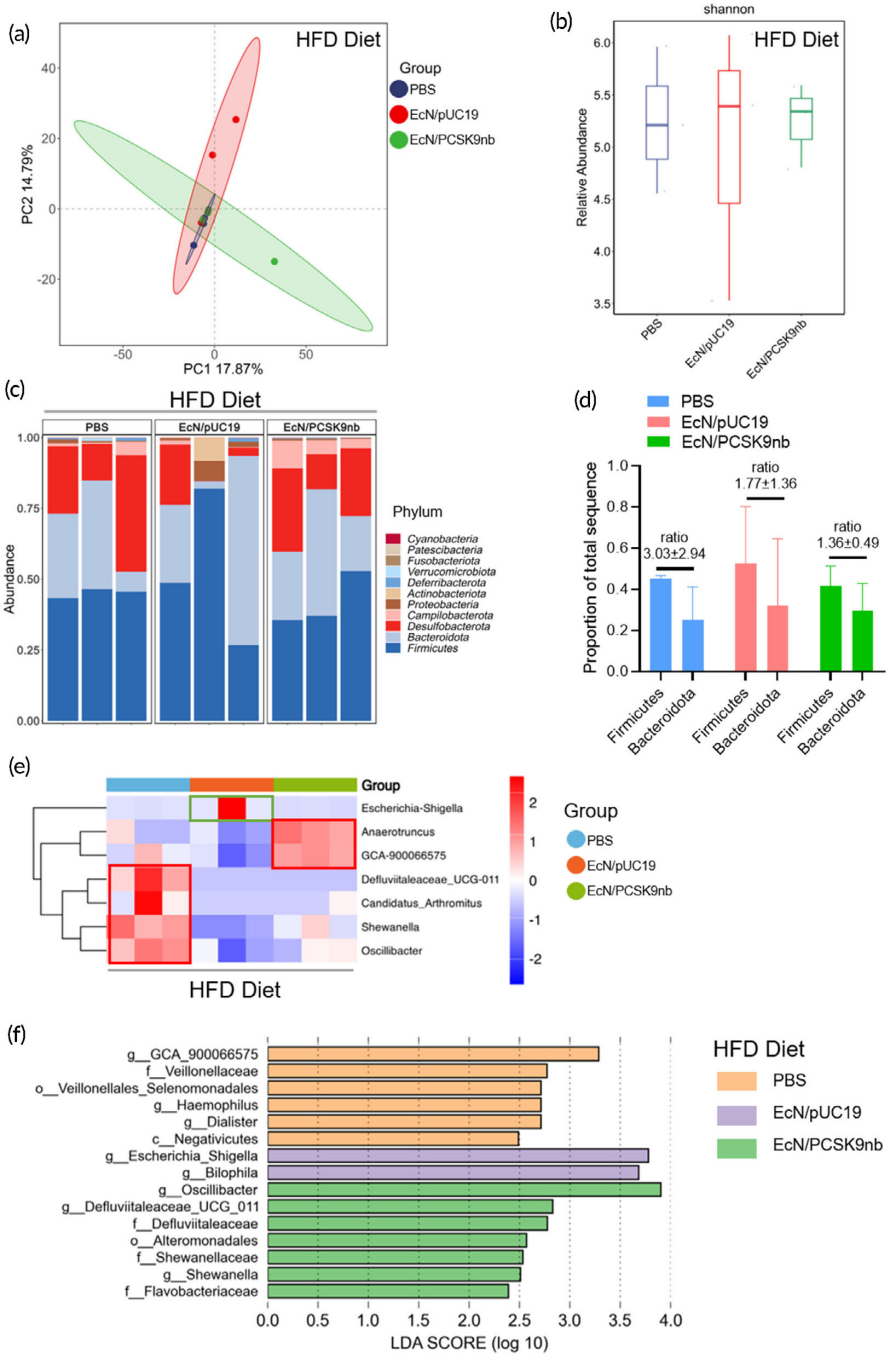
Engineered bacteria regulate gut microbiota diversity and composition. (a) PCoA was conducted to visualize differences in the fecal microbiota structure among the three groups; (b) α‐diversity (Shannon index) of the intestinal microbiota; (c) Characteristics of the community structure of the gut microbiota at the phylum level; (d) Analysis of the *Firmicutes*/*Bacteroidetes* (F/B) ratio in the gut microbiota of mice under different interventions; (e) Differences in the community structure of gut microbiota at the genus level among three groups of mice; (f) Analysis of the differences in the intestinal microbiota by LEfSe (LDA score ≥3, *p* < 0.05).

16S rRNA (V3–V4 region) sequencing was performed to analyze the feces of mice in the HFD group and those intervened with EcN/pUC19 or EcN/PCSK9nb under high‐fat diet conditions. Principal components analysis (PCoA) was performed to visualize variations in fecal microbiota structure among the three groups. As anticipated, there were differences in the fecal microbiota structure between mice in the EcN/PCSK9nb group and those in the other two groups (Figure 7a).

The α‐diversity of the gut microbiota of the three groups of mice (Shannon, Simpson, Goods coverage, observed species, and Chao1 index) showed that EcN/PCSK9nb and EcN/pUC19 were higher than the PBS treatment group, but there is no statistical difference (Figure 7b and Figure S7a–d). The differences in the community structure among the three groups of mice at the phylum and genus levels were compared. *Firmicutes* and *Bacteroidetes* were the dominant phyla in all groups (Figure 7c). In the mice in the EcN/PCSK9nb group, the level of *Firmicutes* tended to decrease. The ratio of *Firmicutes*/*Bacteroidetes* (F/B) in the gut microbiota of three groups of mice: PBS, EcN/pUC19, and EcN/PCSK9nb tended to decrease, with ratios of 3.03 ± 2.94, 1.77 ± 1.36, and 1.36 ± 0.49, respectively (Figure 7d). An elevated F/B ratio is usually positively correlated with obesity, metabolic disorders, and other diseases, and is associated with increased heat extraction from food, fat deposition, lipogenesis, and impaired insulin sensitivity.^38^

Intragastric administration of EcN/PCSK9nb engineered bacteria can reduce the increase in the F/B ratio caused by a high‐fat diet, indicating that EcN/PCSK9nb engineered bacteria can improve metabolic disorders caused by a high‐fat diet by regulating the composition of gut microbiota. At the genus level, *Anaerotruncus* and *GCA‐900066575* were the two genera with the highest relative abundance in the EcN/PCSK9nb group (Figure 7e). Species level analysis showed that *Lachnoclostridium*, *Anaerotruncus*, and *GCA‐900066575* were the most abundant species in the EcN/PCSK9nb group (Figure S7e). *Defluviitaleaceae_UCG‐011*, *Candidatus_Arthromitus*, *Shewanella*, and *Oscillibacter* dominated in the PBS group. Linear discriminant analysis effect size (LefSE) analysis of the biome differences among the three groups also showed that GCA‐900066575 was dominant in EcN/PCSK9nb, indicating that it had important biological functions (Figure 7f).

### 3.8 Comparative analysis of the therapeutic effects of EcN/PCSK9nb and atorvastatin

**FIGURE 8:**
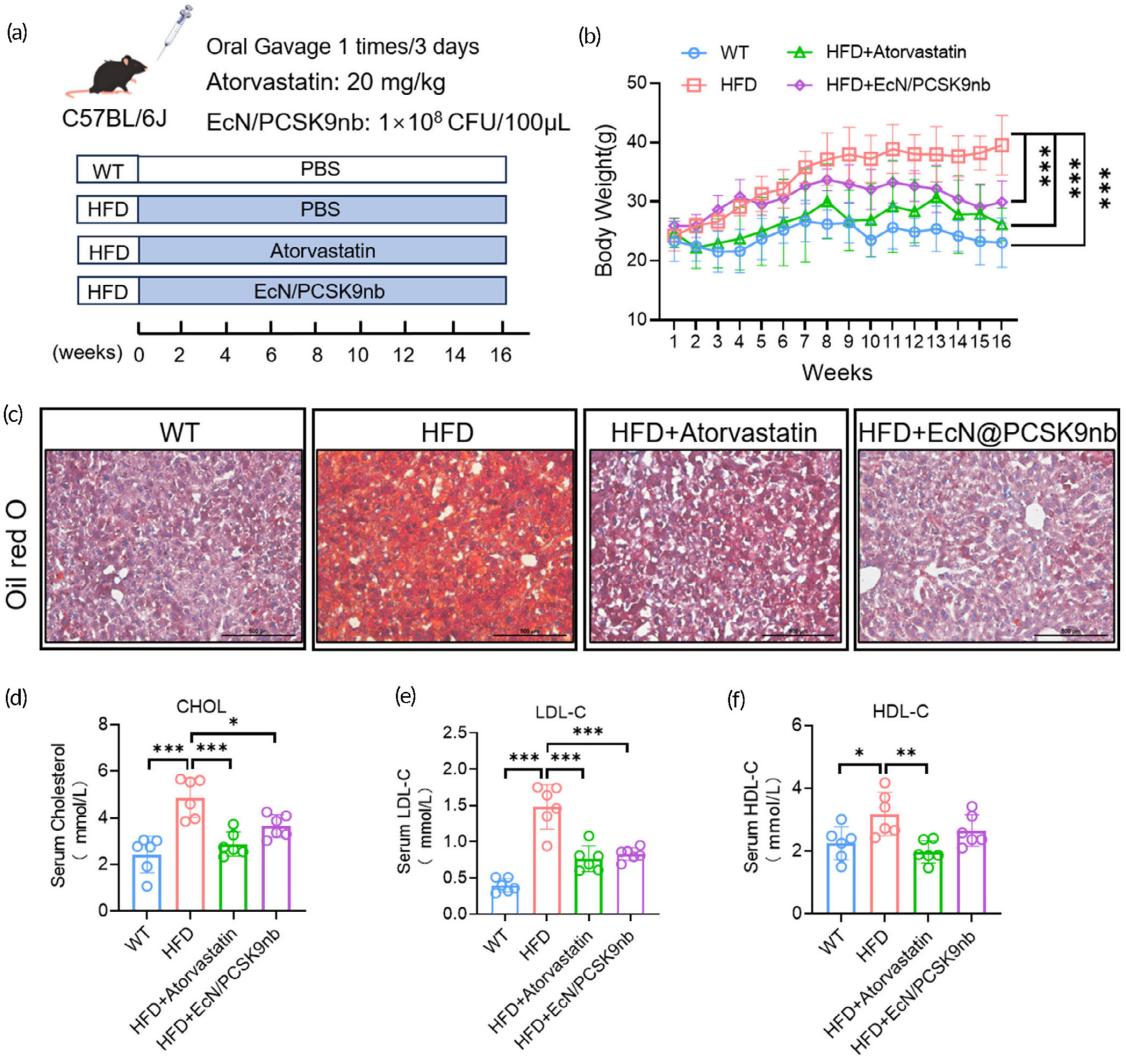
Comparative analysis of the therapeutic effects of EcN/PCSK9nb and atorvastatin. (a) Experimental scheme of drug administration in mice; (b) Body weight change curves of mice, statistical data presented as mean ± SD (*n* = 6); (c) Liver tissue section stained with Oil Red O, scale bar: 500 μm; (d–f) Serum CHOL, LDL‐C, and HDL‐C levels displayed as mean ± SD (*n* = 6). *p < 0.05, **p < 0.01, ***p < 0.001, ****p < 0.0001.

To assess whether there was a difference in the therapeutic efficacy of the engineered strain, EcN/PCSK9nb, and the existing clinically used lipid‐lowering drugs, we administered EcN/PCSK9nb (1 × 10^8^ CFU/100 μL) and atorvastatin (20 mg/kg) orally using gastric gavage every 3 days while mice were fed high‐fat diets (Figure 8a). The body weights of the mice were continuously monitored for 16 weeks, and the body weights of the mice fed EcN/PCSK9nb and atorvastatin were substantially lower than those of the HFD group (Figure 8b). Oil Red O staining of the livers of the mice showed that the number and area of red lipid droplets in the livers of the mice fed atorvastatin and EcN/PCSK9nb were substantially reduced compared to those of the mice fed the HFD (Figure 8c). The serum levels of total cholesterol, LDL‐C, and HDL‐C in each group of mice were analyzed; the oral administration of atorvastatin and EcN/PCSK9nb substantially lowered serum total cholesterol levels, and both had the same LDL‐C lowering effect; however, mice that had orally administered atorvastatin also had substantially lower HDL‐C levels (Figure 8d–f). These results consistently suggest that EcN/PCSK9nb engineered bacteria have therapeutic effects similar to those of atorvastatin.

## 4 DISCUSSION

In the current study, we focused on the construction, physiological traits, and biocompatibility of the engineered bacterium, EcN/PCSK9nb, and the treatment of hyperlipidemia and atherosclerosis in mouse models. Based on previous studies, we codon‐optimized the amino acid sequence of the previously reported high‐affinity fragment, VHH‐B11, of the human PCSK9 nanobody (hPCSK9nb) prepared by alpaca to simplify the prokaryotic expression system. Moreover, secreted PCSK9nb was shown to have the ability to bind to mouse‐derived PCSK9 in vitro using a Co‐IP assay, which provided us with direct evidence that PCSK9nb secreted by EcN/PCSK9nb engineered bacteria may play a role in lowering LDL‐C in vivo and reducing lipid accumulation in the liver. Since PCSK9 is highly conserved in mammals, this also lays the foundation for further translational medicine research in the future with EcN/PCSK9nb engineered bacteria.

In this study, EcN/PCSK9nb engineered bacteria could normally express and secrete PCSK9nb in vivo and in vitro; PCSK9nb could also enter the body through the intestinal barrier and was found to be enriched in the liver, spleen, and kidneys, whereas no large‐scale distribution was detected in other major organs. In addition, the assay for hepatic inflammatory factor levels showed a decrease in the levels of the relevant inflammatory factors in the EcN/PCSK9nb group, indicating the advantages of expressing secreted PCSK9nb in terms of biocompatibility and inflammatory response.

We evaluated the therapeutic effects of EcN/PCSK9nb engineered bacteria in murine models of hyperlipidemia and atherosclerosis. These results confirmed that EcN/PCSK9nb engineered bacteria could alleviate the development of atherosclerosis by protecting LDLR, substantially reducing serum LDL‐C levels and hepatic lipid accumulation, and reducing inflammation. C57BL/6J mice treated with EcN/PCSK9nb showed a marked decrease in serum HDL‐C levels, based on a previous study showing that apolipoprotein E (APOE) carries HDL and binds to upregulated LDLR in *PCSK9−/−* mice.^39^ This may explain the decreased HDL‐C levels in the serum of C57BL/6J mice gavaged with EcN/PCSK9nb engineered bacteria, whereas we also observed that there was no significant change in HDL‐C levels in *ApoE−/−* mice.

The control strain, EcN/pUC19, exhibited some ameliorative effects when administered alone to mice, which we hypothesized was related to the probiotic properties of our chosen chassis organism, EcN. The probiotic properties of EcN (such as antibacterial activity, immunomodulation, metabolite secretion, etc.) may improve metabolic abnormalities under HFD via multiple pathways: for example, EcN inhibits the excessive proliferation of other *Enterobacteriaceae* by secreting antibacterial proteins (such as microcins), thereby improving gut microbiota imbalance caused by HFD.^40^ HFD can induce metabolic inflammation. Natural EcN improves HFD‐related intestinal leakage and systemic inflammation by secreting immunomodulatory factors (such as TGF‐β) or promoting the expression of intestinal barrier proteins, thereby alleviating host metabolic inflammation.^41^ In addition, certain secondary metabolites of EcN (such as colibactin) may participate in host physiological regulation by regulating host immunity or microbial interactions.^42^

In the HFD group, the main composition of gut microbiota in the EcN/pUC19 group was different from that in the PBS and EcN/PCSK9nb groups in this study. For instance, the abundance of dominant species, such as *Desulfovibrio*, *Candidatus_Arthromitus*, *Shewanella*, *Alloprevotella*, *Bacteroides*, *Negativibacillus*, and *Oscillibacter*, was reduced in the PBS group. Compared to the dominant species, *Lachnoclostridium*, *GCA‐900066575*, and *Anaerotruncus* in the EcN/PCSK9nb group, the relative abundance of the EcN/pUC19 group was similarly reduced, but the level of *Escherichia‐Shigella* increased (Figure S7c). Existing studies have shown that metabolic disorders are closely associated with changes in the intestinal microbiota. Intervention with specific drugs or compounds (such as betaine or baicalein) in HFD mice can effectively alleviate HFD‐induced obesity, improve metabolic disorders, and remodel the intestinal microbiota.^43^, ^44^, ^45^ The increase in relative abundance of *Lachnoclostridium*, a beneficial gut bacterium related to immunomodulation,^46^, ^47^, ^48^ dietary fiber metabolism, and particularly SCFAs production,^49^ may be associated with the above phenomena. This is also consistent with the characteristics of intestinal microbiota changes in mice intervened by EcN/PCSK9nb in this study. Taken together, these results suggest that the probiotic properties of EcN itself, as well as the characteristic alterations in the structure of the microbiota it induces, may be one of the reasons for the ability of the EcN group to reduce serum LDL‐C and hepatic lipid accumulation to some extent.

Although the current study indicates that the constructed EcN/PCSK9nb engineered bacterium demonstrates a good ability to lower LDL‐C levels and mitigate atherosclerosis, as well as a natural advantage in controlling costs owing to properties such as ease of cultivation, there are also some issues that need to be addressed. For example, how can a bacteriophage‐expressing PCSK9nb be secreted extracellularly more efficiently? To address this issue, we tried to screen signal peptide sequences from different sources (*PhoAss*, *DsbAss*, *PelBss*, *ProAss*) during our experiments,^50^ and we also tried to develop the use of the enterohemorrhagic *E. coli* type O157 (EHEC O157) α‐hemolysin secretion system (HlyABD) to release expressed PCSK9nb directly into the extracellular space bypassing the periplasmic space (data not shown).^51^ EcN/PCSK9nb could only be retained in mice for approximately 4 days in in vivo colonization experiments; thus, intermittent continuous dosing is required for the actual administration of EcN/PCSK9nb. The gut microbiota mediates colonization resistance through direct mechanisms like nutrient/space competition, bacteriocin secretion, and inhibitory metabolites (e.g., SCFAs, secondary BAs), and indirect pathways such as mucosal barrier reinforcement and immune activation (e.g., RegIIIγ, IL‐22).^52^ EcN's poor gut colonization in mice may stem from insufficient nutrient utilization against native microbes, susceptibility to microbiota‐produced bacteriocins and host antimicrobials (e.g., RegIIIγ), weak epithelial adhesion, and immune‐mediated clearance. Moreover, we also speculate that this may be related to the fact that EcN itself is derived from the human intestine and has insufficient adaptability to the environment in mice.^53^ Replacing chassis organisms such as intestinal *Bacteroidetes* may help improve the intestinal colonization of engineered bacteria.^54^ In addition, constructing nutrient‐deficient EcN strains and realizing the integration of PCSK9nb into the genome, thus reducing the risk of spreading antibiotic resistance genes and enhancing the stability of expression, these will be our next directions for further improvement.

## 5 CONCLUSIONS

In this study, we successfully constructed a strain of engineered bacterium EcN/PCSK9nb that can be used orally and is effective in lowering LDL‐C levels, reducing hepatic lipid accumulation, attenuating inflammation, and alleviating atherosclerosis. These results showed that the engineered bacterium can ameliorate hyperlipidemia and atherosclerosis. The research results show important translational significance and application value in reducing the cost of clinical medication, improving the route of drug delivery, increasing patient compliance, etc., and provide new ideas and an experimental basis for the development of PCSK9 inhibitor drugs.

## AUTHOR CONTRIBUTIONS

Z.L. and H.T. conceived the study, designed experiments, and supervised the research. Z.L., J.Y.X., and C.S. wrote the manuscript. C.W., Y.S.G., X.R.C., Z.F.N., X.H.M., X.K.Q., X.L.C., and D.D.J. performed the experiments. H.Z. and C.S. provided the critical experimental materials and contributed to the data discussion. Y.C.S. provided technical support for sequencing data analysis.

## CONFLICT OF INTEREST STATEMENT

The authors declare no conflict of interest.

## Supporting information


**Figure S1.** Conservation Analysis of PCSK9 in Humans and Mice. (a) Comparison of amino acid sequences of PCSK9 between humans and mice; (b) Analysis of structural domains of PCSK9 between humans and mice; (c) Comparison of the tertiary structures of PCSK9 between humans and mice; (d) Gene map of recombinant plasmid pUC19‐Tac‐NSP4‐PCSK9nb‐HA.
**Figure S2.** The effect of NSP4 signal peptide on the expression and secretion efficiency of PCSK9nb by EcN/PCSK9nb was detected by Western Blot.
**Figure S3.** Randomly selected flat plate colonies were subjected to primer‐specific PCR validation.
**Figure S4.** Immunofluorescence analysis of HA‐tag expression in heart, liver, spleen, lung and kidney of engineered bacteria EcN/PCSK9nb. The EcN/pUC19 treatment group served as the negative control. Red represents the HA‐tag, blue represents DAPI, scale: 50 μm.
**Figure S5.** Effects of oral EcN/PCSK9nb engineering bacteria on serum CHOL, LDL‐C and HDL‐C levels at 4 (a) and 8 (b) weeks.
**Figure S6.** (a) The statistical results of Oil Red O staining in the liver; (b) LDL‐R levels of *ApoE*−/− mice livers were evaluated by WB.
**Figure S7.** Analysis of 16 s rRNA amplicon sequencing results. (a–d) α‐diversity (Simpson, goods_coverage, observed species and chao1 index) of the intestinal microbiota; (c) Heatmap of differences in the community structure of gut microbiota at the species level among three groups of mice.
**Table S1.** Genetic information of recombinant plasmid.
**Table S2.** Primers for realtime‐qPCR in this study.

## Data Availability

The 16S rRNA sequencing data from this study has been submitted to the NCBI Sequence Read Archive (https://www.ncbi.nlm.nih.gov/sra) under accession number PRJNA1171950.
