# Supplementary material for: Engineered probiotic ameliorates hyperlipidemia and atherosclerosis by secreting PCSK9 nanobodies and regulating gut microbiota
Source: Bioeng Transl Med. 2025 Sep 19;10(6):e70076. doi: 10.1002/btm2.70076 (PMC12617544; doi:10.1002/btm2.70076)
Supplement: Supplementary file 1 — Figure S1. Conservation Analysis of PCSK9 in Humans and Mice. (a) Comparison of amino acid sequences of PCSK9 between humans and mice; (b) Analysis of structural domains of PCSK9 between humans and mice; (c) Comparison of the tertiary structures of PCSK9 between humans and mice; (d) Gene map of recombinant plasmid pUC19‐Tac‐NSP4‐PCSK9nb‐HA. Figure S2. The effect of NSP4 signal peptide on the expression and secretion efficiency of PCSK9nb by EcN/PCSK9nb was detected by Western Blot. Figure S3. Randomly selected flat plate colonies were subjected to primer‐specific PCR validation. Figure S4. Immunofluorescence analysis of HA‐tag expression in heart, liver, spleen, lung and kidney of engineered bacteria EcN/PCSK9nb. The EcN/pUC19 treatment group served as the negative control. Red represents the HA‐tag, blue represents DAPI, scale: 50 μm. Figure S5. Effects of oral EcN/PCSK9nb engineering bacteria on serum CHOL, LDL‐C and HDL‐C levels at 4 (a) and 8 (b) weeks. Figure S6. (a) The statistical results of Oil Red O staining in the liver; (b) LDL‐R levels of ApoE−/− mice livers were evaluated by WB. Figure S7. Analysis of 16 s rRNA amplicon sequencing results. (a–d) α‐diversity (Simpson, goods_coverage, observed species and chao1 index) of the intestinal microbiota; (c) Heatmap of differences in the community structure of gut microbiota at the species level among three groups of mice. Table S1. Genetic information of recombinant plasmid. Table S2. Primers for realtime‐qPCR in this study. [file BTM2-10-e70076-s001.docx]

**Supplementary Materials**
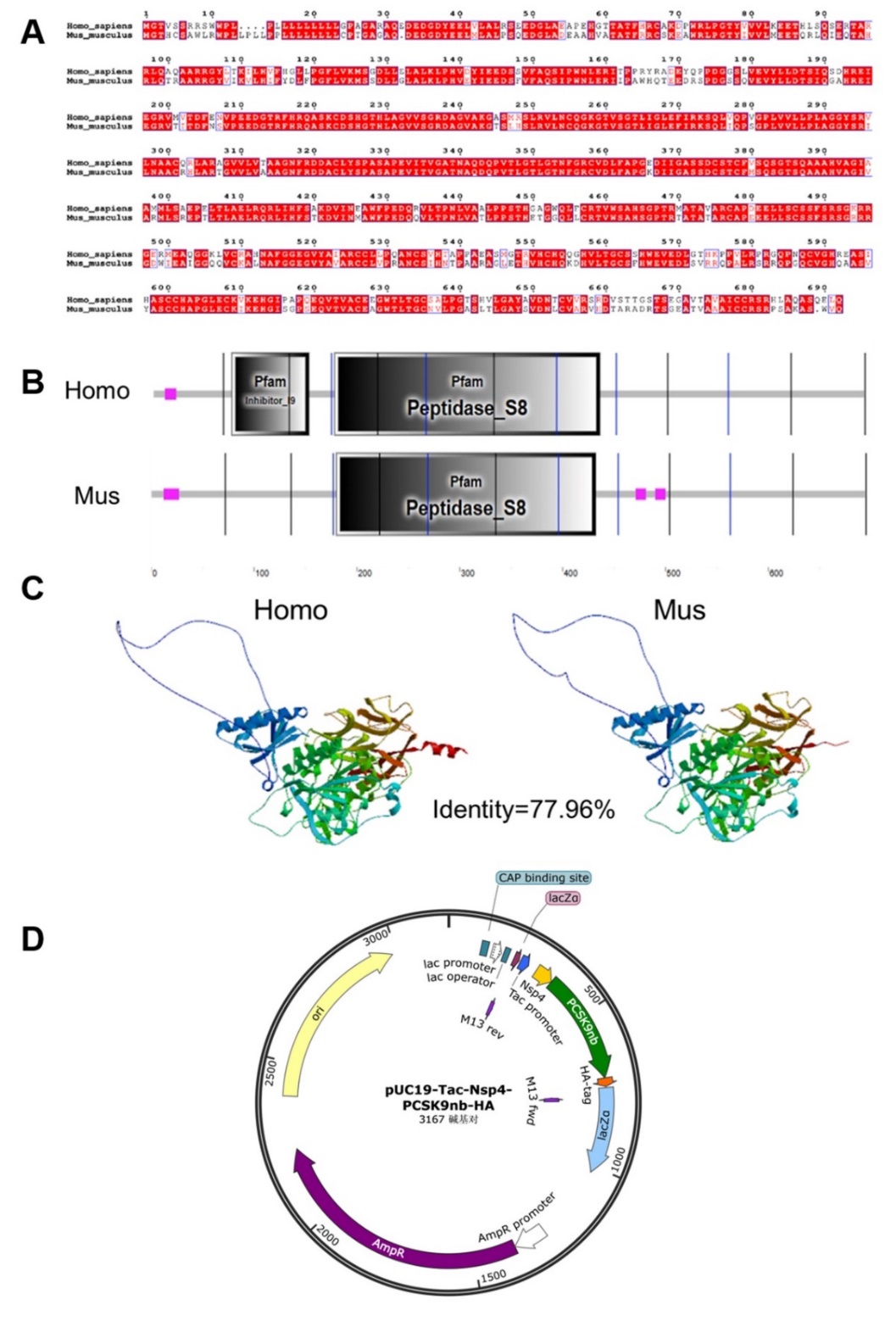


Figure S1. Conservation Analysis of PCSK9 in Humans and Mice. (A) Comparison of amino acid sequences of PCSK9 between humans and mice; (B) Analysis of structural domains of PCSK9 between humans and mice; (C) Comparison of the tertiary structures of PCSK9 between humans and mice; (D) Gene map of recombinant plasmid pUC19-Tac-NSP4-PCSK9nb-HA.


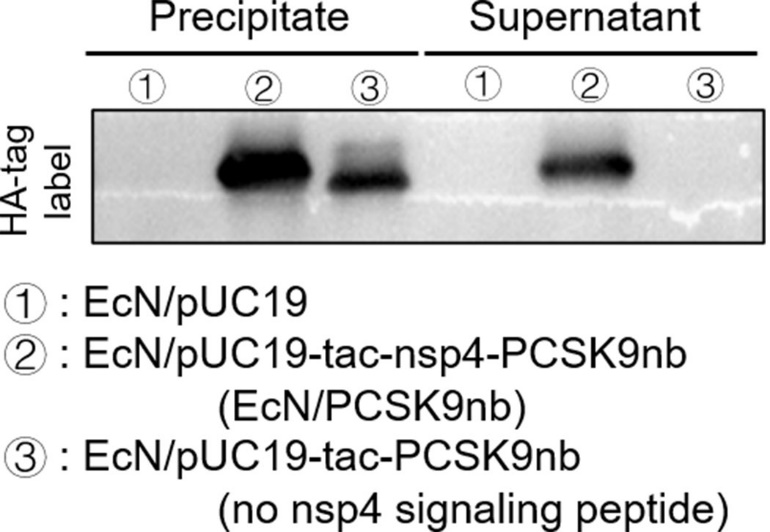


Figure S2. The effect of NSP4 signal peptide on the expression and secretion efficiency of PCSK9nb by EcN/PCSK9nb was detected by Western Blot.


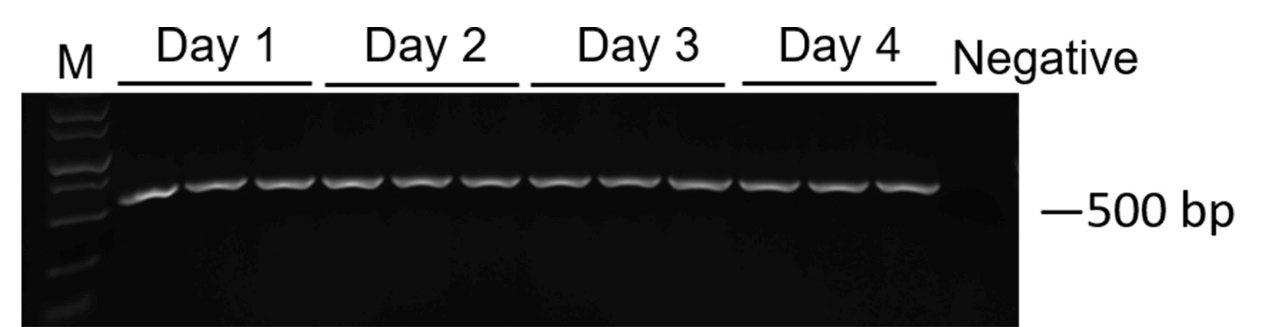


Figure S3. Randomly selected flat plate colonies were subjected to primer-specific PCR validation.


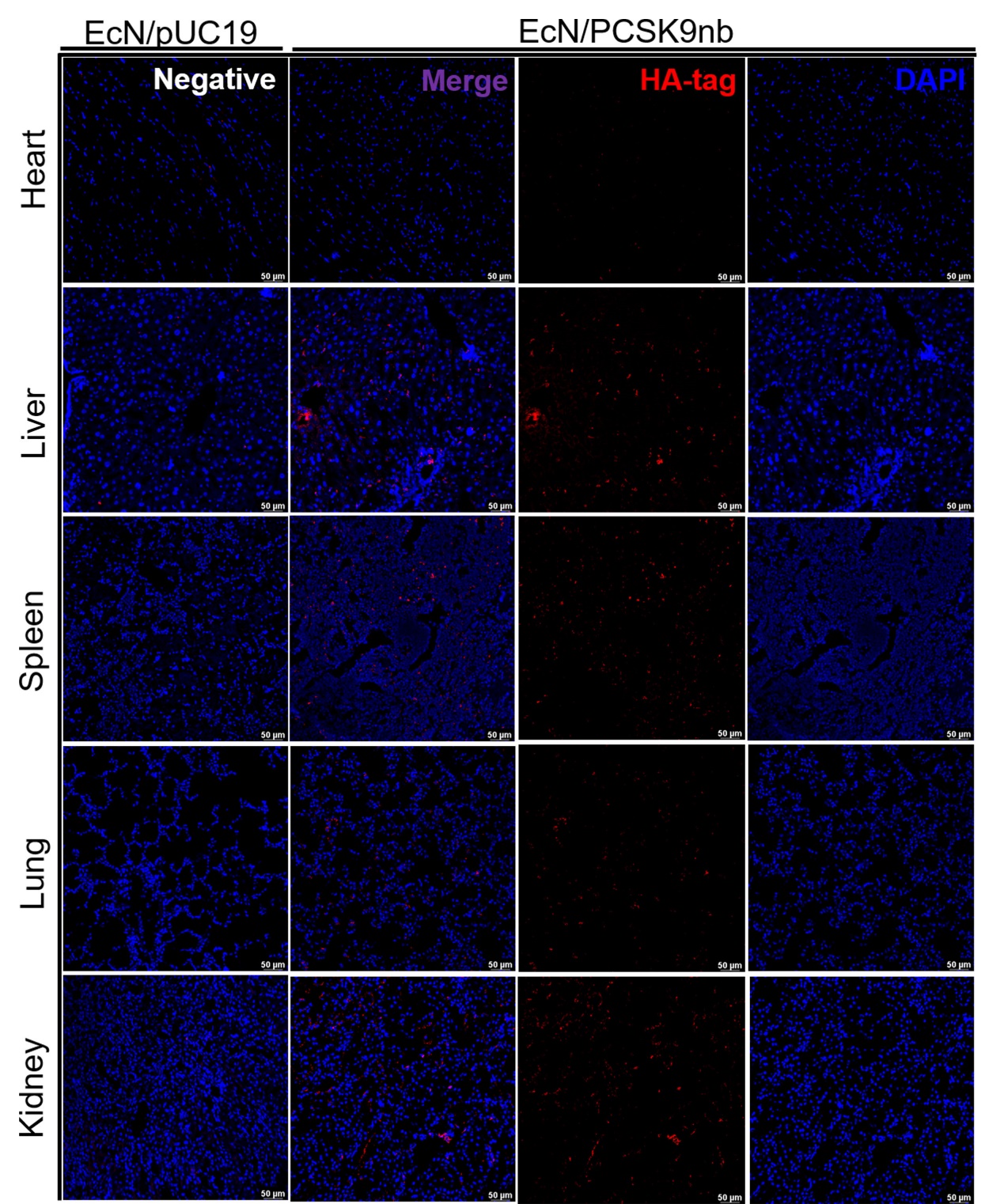


Figure S4. Immunofluorescence analysis of HA-tag expression in heart, liver, spleen, lung and kidney of engineered bacteria EcN/PCSK9nb. The EcN/pUC19 treatment group served as the negative control. Red represents the HA-tag, blue represents DAPI, scale: 50 μm.


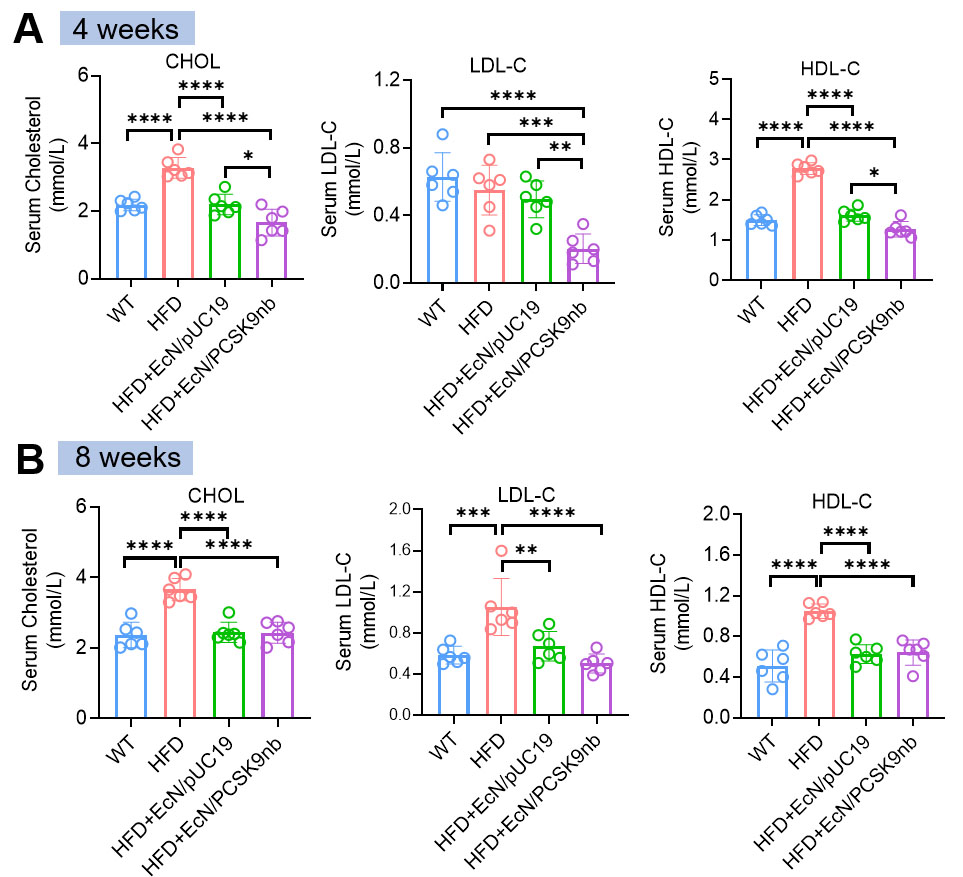
Figure S5. Effects of oral EcN/PCSK9nb engineering bacteria on serum CHOL, LDL-C and HDL-C levels at 4 (A) and 8 (B) weeks.


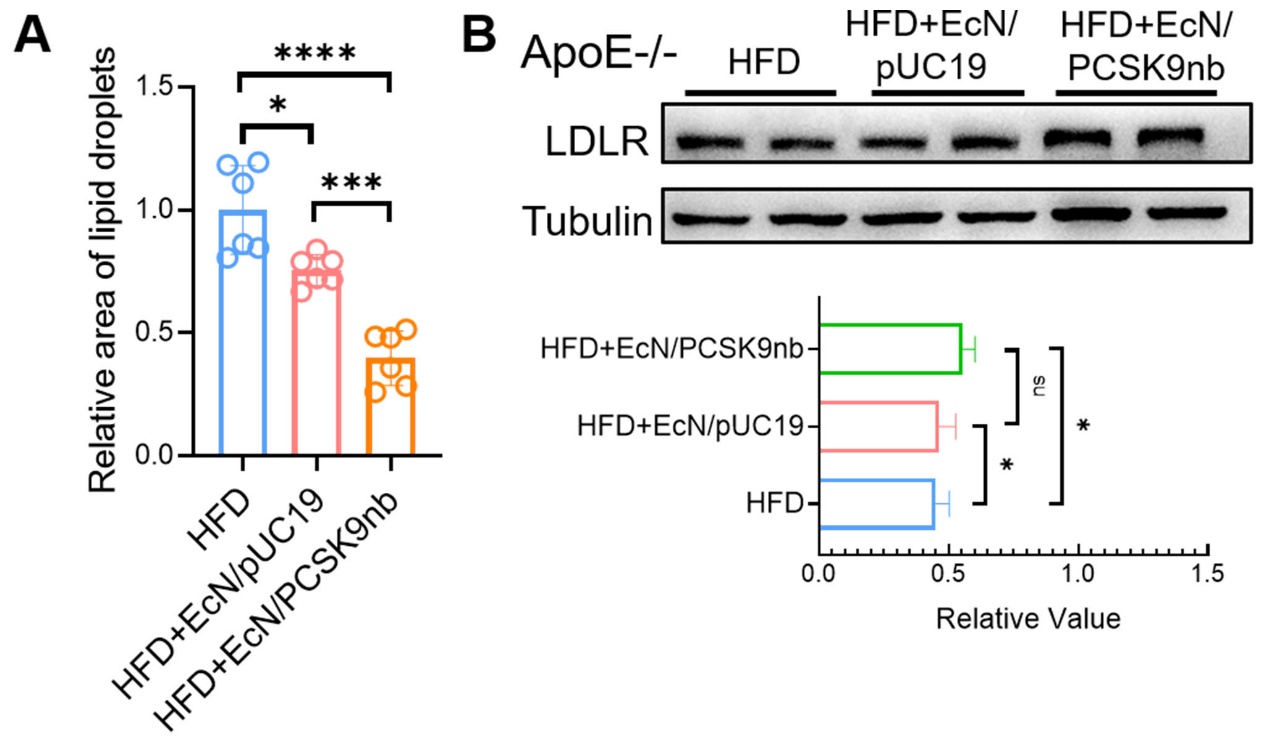


Figure S6. (A) The statistical results of Oil Red O staining in the liver; (B) LDL-R levels of *ApoE*-/- mice livers were evaluated by WB.


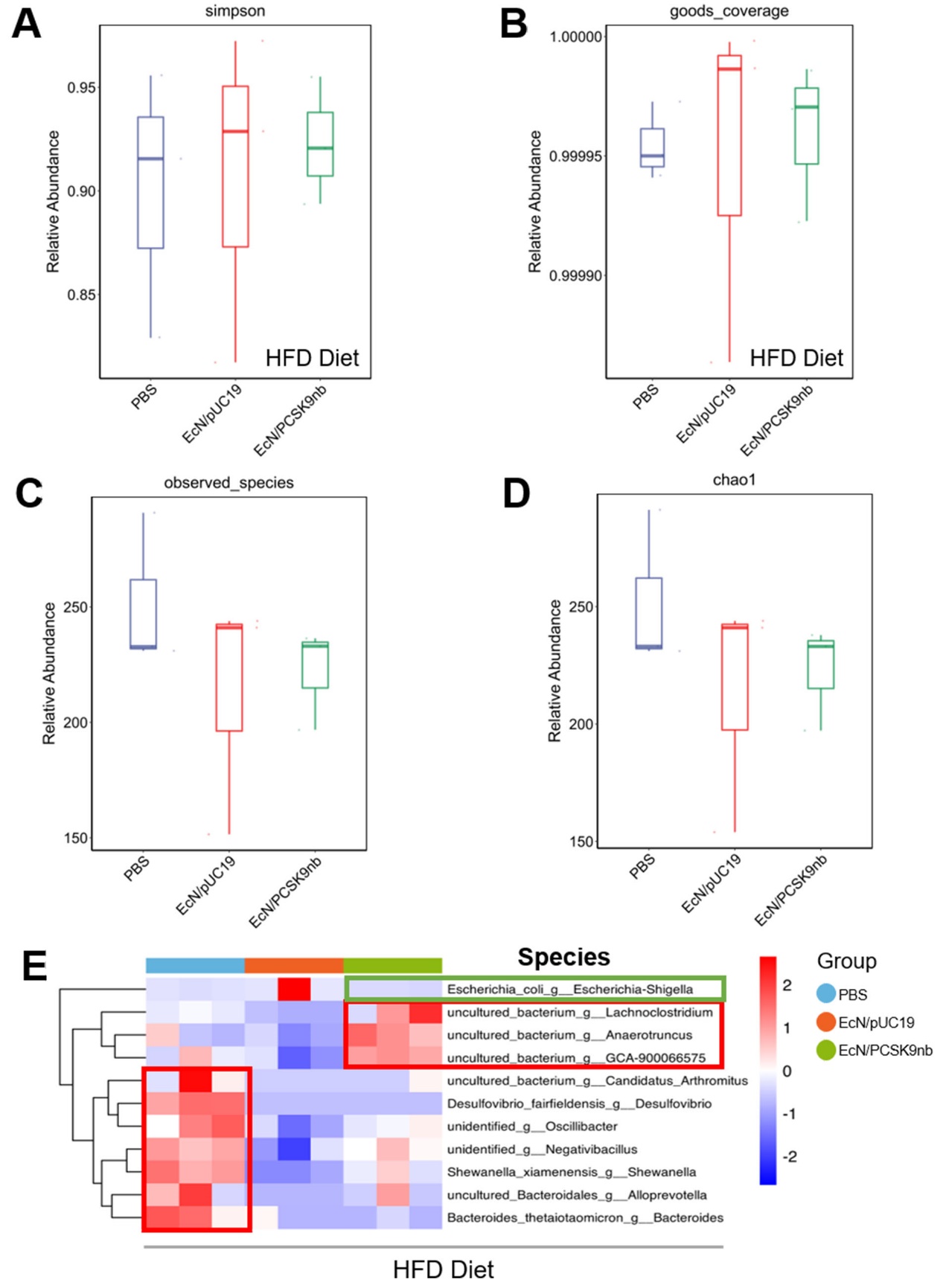


Figure S7. Analysis of 16s rRNA amplicon sequencing results. (A-D) α-diversity (Simpson, goods_coverage, observed species and chao1 index) of the intestinal microbiota; (C) Heatmap of differences in the community structure of gut microbiota at the species level among three groups of mice.

Table S1. Genetic information of recombinant plasmid

| **Elements** | **Sequence (5‘-3’)** | **Located**  **(5‘-3’)** |
| --- | --- | --- |
| VHH-B11(Amino acids sequence) | EVQLVESGGGSVQAGGSLRLSCTVSGYTYSSNCMGWFRQAPGKEHEGVASIYIGGGSTYYADSVKGRFTISQDNAKNTVYLQMNSLKPEDTAMYYCAVGCQGLVDFGYWDQGTQVTVSS |  |
| The full length of synthetic genetic information | AAGCTTTTGACAATTAATCATCGGCTCGTATAATGTGTGGAATTTCACACAGGAAACAGAATTCATGAAAAAGATTACCGCTGCTGCTGGTCTGCTGCTCCTCGCTGCCCAGCCGGCGATGGCGGAAGTGCAACTGGTGGAGAGCGGTGGTGGTAGTGTGCAAGCGGGTGGCAGCCTGCGCCTGAGCTGCACCGTGAGCGGCTATACCTATAGCAGCAACTGCATGGGCTGGTTTCGCCAAGCGCCGGGCAAAGAACATGAAGGCGTGGCGAGCATTTATATTGGCGGTGGCAGTACCTATTATGCGGATAGCGTGAAAGGCCGCTTTACCATTAGCCAAGATAACGCGAAAAACACCGTGTATCTGCAGATGAACAGCCTGAAACCGGAAGATACCGCGATGTATTATTGCGCGGTGGGCTGCCAAGGCCTGGTGGATTTTGGCTATTGGGATCAAGGCACCCAAGTGACCGTGAGCAGCTACCCATACGATGTTCCAGATTACGCTTAAGGATCC | 1-517 |
| Hind III | AAGCTT | 1-6 |
| tac promoter | TTGACAATTAATCATCGGCTCGTATAATG | 7-35 |
| RBS | AGGAAA | 51-56 |
| NSP4 signal peptide | ATGAAAAAGATTACCGCTGCTGCTGGTCTGCTGCTCCTCGCTGCCCAGCCGGCGATGGCG | 65-124 |
| VHH-B11  (The nucleotide sequence after codon optimization) | GAAGTGCAACTGGTGGAGAGCGGTGGTGGTAGTGTGCAAGCGGGTGGCAGCCTGCGCCTGAGCTGCACCGTGAGCGGCTATACCTATAGCAGCAACTGCATGGGCTGGTTTCGCCAAGCGCCGGGCAAAGAACATGAAGGCGTGGCGAGCATTTATATTGGCGGTGGCAGTACCTATTATGCGGATAGCGTGAAAGGCCGCTTTACCATTAGCCAAGATAACGCGAAAAACACCGTGTATCTGCAGATGAACAGCCTGAAACCGGAAGATACCGCGATGTATTATTGCGCGGTGGGCTGCCAAGGCCTGGTGGATTTTGGCTATTGGGATCAAGGCACCCAAGTGACCGTGAGCAGC | 125-481 |
| HA tag | TACCCATACGATGTTCCAGATTACGCTTAA | 482-511 |
| BamH I | GGATCC | 512-517 |

Table S2. Primers for realtime-qPCR in this study

| **Gene** | **Forward Promer 5’-3’** | **Reverse Primer 5’-3’** |
| --- | --- | --- |
| *Il-1β* | TCTTTGAAGTTGACGGACCC | TGAGTGATACTGCCTGCCTG |
| *Il-6* | AGAAGGAGTGGCTAAGGACCAA | AACGCACTAGGTTTGCCGAGTA |
| *β-Actin* | GTGACGTTGACATCCGTAAAGA | GCCGGACTCATCGTACTCC |
| *Mcp-1* | ATGGCCTCCCTCTCATCAGT | ACCTTAGGGCAGATGCAGTTTTA |
| *Tnf-α* | GCAACTGCTGCACGAAATC | CTGCTTGTCCTCTGCCCAC |
